# Gestational Exposure to Phthalates and Social Responsiveness Scores in Children Using Quantile Regression: The EARLI and HOME Studies

**DOI:** 10.3390/ijerph18031254

**Published:** 2021-01-30

**Authors:** Marisa A. Patti, Craig Newschaffer, Melissa Eliot, Ghassan B. Hamra, Aimin Chen, Lisa A. Croen, M. Daniele Fallin, Irva Hertz-Picciotto, Geetika Kalloo, Jane C. Khoury, Bruce P. Lanphear, Kristen Lyall, Kimberly Yolton, Joseph M. Braun

**Affiliations:** 1 Department of Epidemiology, Brown University, Providence, RI 02903, USA; Melissa_Eliot@brown.edu (M.E.); joseph_braun_1@brown.edu (J.M.B.); 2 A.J. Drexel Autism Institute, Drexel University, Philadelphia, PA 19104, USA; newschaffer@psu.edu (C.N.); kld98@drexel.edu (K.L.); 3 College of Health & Human Development, Pennsylvania State University, State College, PA 16801, USA; 4 Department of Epidemiology, Johns Hopkins University, Baltimore, MD 21205, USA; ghassanhamra@jhu.edu; 5 Department of Biostatistics Epidemiology and Informatics, University of Pennsylvania, Philadelphia, PA 19104, USA; Aimin.Chen@pennmedicine.upenn.edu; 6 Division of Research, Kaiser Permanente Northern California, Oakland, CA 94612, USA; lisa.a.croen@kp.org; 7 Department of Mental Health, Johns Hopkins University, Baltimore, MD 21205, USA; dfallin@jhu.edu; 8 Department of Public Health Sciences, University of California, Davis, CA 95616, USA; iher@ucdavis.edu; 9 HealthCore Inc., Wilmington, DE 19801, USA; geetika.kalloo@gmail.com; 10 Division of Biostatistics and Epidemiology, Cincinnati Children’s Hospital Medical Center, Cincinnati, OH 45267, USA; jane.Khoury@cchmc.org; 11 Department of Pediatrics, University of Cincinnati College of Medicine, Cincinnati, OH 45267, USA; kimberly.yolton@cchmc.org; 12 Faculty of Health Sciences, Simon Fraser University, Vancouver, BC, Canada; bruce_lanphear@sfu.ca; 13 Department of Pediatrics, Cincinnati Children’s Hospital Medical Center, Cincinnati, OH 45267, USA

**Keywords:** phthalates, prenatal, endocrine-disrupting chemicals, neurodevelopment

## Abstract

Linear regression is often used to estimate associations between chemical exposures and neurodevelopment at the mean of the outcome. However, the potential effect of chemicals may be greater among individuals at the ‘tails’ of outcome distributions. Here, we investigated distributional effects on the associations between gestational phthalate exposure and child Autism Spectrum Disorder (ASD)-related behaviors using quantile regression. We harmonized data from the Early Autism Risk Longitudinal Investigation (EARLI) (*n* = 140) Study, an enriched-risk cohort of mothers who had a child with ASD, and the Health Outcomes and Measures of the Environment (HOME) Study (*n* = 276), a general population cohort. We measured concentrations of 9 phthalate metabolites in urine samples collected twice during pregnancy. Caregivers reported children’s ASD-related behaviors using the Social Responsiveness Scale (SRS) at age 3–8 years; higher scores indicate more ASD-related behaviors. In EARLI, associations between phthalate concentrations and SRS scores were predominately inverse or null across SRS score quantiles. In HOME, positive associations of mono-n-butyl phthalate, monobenzyl phthalate, mono-isobutyl phthalate, and di-2-ethylhexyl phthalate concentrations with SRS scores increased in strength from the median to 95th percentile of SRS scores. These results suggest associations between phthalate concentrations and SRS scores may be stronger in individuals with higher SRS scores.

## 1. Introduction

Phthalates are a class of synthetic chemicals used in some personal care products, plastics, adhesives, building materials, and food processing [1,2,3,4,5]. Phthalates are readily released into the environment [6,7], exposing humans through ingestion, inhalation, and dermal absorption [8,9,10]. Phthalates are quickly excreted from the body [11,12,13], but are measurable in most humans because exposure is ubiquitous and chronic [14,15]. As endocrine disrupting chemicals (EDCs) that can cross the placenta [16,17], phthalates may influence thyroid hormones and testosterone [18,19,20,21,22,23,24,25], which are essential to fetal neurodevelopment in both animals and humans [26,27,28,29,30,31,32].

Gestation is a critical period of fetal brain development, and environmental insults during this period of heightened vulnerability have been linked to atypical social behaviors in childhood [33]. Previous reports have identified heterogeneity of the association between urinary phthalate concentrations and atypical social behaviors in childhood. While some studies have identified an inverse relationship between prenatal phthalate concentrations and behavior traits associated with Autism Spectrum Disorder (ASD) in childhood [34,35,36], others report null findings [37,38,39,40]. However, these inconsistent findings may depend on the source population and method of assessing ASD related traits, presence and potential of ASD related risk factors, and neurodevelopmental outcomes measured. For example, in a large, general population, pan-Canadian cohort study, gestational phthalate concentrations were associated with more autistic traits in children [41]. However, in an enriched risk cohort of mothers who previously had a child with autism, which measured phthalate metabolites from three different timepoints during gestation, found inverse and null associations between phthalates and autistic diagnoses [39].

Linear regression is typically used to estimate the mean difference in neurodevelopmental outcomes across distributions of phthalate exposure. However, linear regression does not identify exposure-associated differences among participants at the tails of these distributions. This may be important to consider if the effects of phthalates differ across subsets of the population based on susceptibility to neurotoxicants, where increased susceptibility is indicated by higher SRS scores. This may be one reason why some previous studies have not identified associations of prenatal phthalate exposure with ASD-related traits. As a solution, quantile regression is a statistical technique that estimates exposure-outcome associations at different percentiles of the outcome, allowing for characterization of associations across the full spectrum of an outcome distribution, including those who may be most susceptible [42,43,44]. Thus, quantile regression can capture differences in susceptibility to exposures, where higher quantiles of an SRS score serve as a marker of susceptibility.

Here, we expand the work of previous studies by using quantile regression to assess the relation between gestational urinary phthalate concentrations and behavior traits related to ASD in children using mother-child pairs from the Early Autism Risk Longitudinal Investigation study (EARLI, *n* = 140) and from the Health Outcomes and Measure of the Environment Study (HOME, *n* = 276). We hypothesize that urinary concentrations of phthalate metabolites during pregnancy will be more strongly associated with ASD related traits among participants with higher social responsiveness scores.

## 2. Materials and Methods

### 2.1. Participants

We used data from two prospective pregnancy cohorts, the EARLI and HOME studies. EARLI is an enriched-risk cohort of women who previously had a child diagnosed with ASD. Recruitment details and information regarding data collection have been previously published [45]. Briefly, from 806 eligible pregnant women and their biological child with ASD, 264 women were recruited between 2009 and 2012. Recruitment took place from four sites in the United States (US): Pennsylvania (Drexel/Children’s Hospital of Philadelphia), Maryland (Johns Hopkins/Kennedy Krieger Institute), and Northern California (UC Davis and Northern California Kaiser Permanente). Each participating site’s Institutional Review Board (IRB) approved the EARLI study. To be eligible for participation in EARLI, women must have previously had a child with ASD confirmed by an EARLI clinician, be at least 18 years old, less than 29 weeks gestation, communicative in English or Spanish, and live within 2 h of a study site. Informed, written consent was provided from all women and for their children. From those recruited, 176 women delivered live born, singleton infants. Our analysis included 140 women after we excluded those without covariate information (*n* = 36) (Figure S1).

The HOME Study is a general population, prospective pregnancy and birth cohort study. Information regarding recruitment and data collection details have been previously published [46]. Briefly, we recruited 468 pregnant women from 1263 eligible women attending one of nine prenatal clinics affiliated with three delivery hospitals in the greater Cincinnati, Ohio area between 2003 and 2006. Inclusion criteria specified women be at least 18 years old, 16 ± 3 weeks gestation, Human Immunodeficiency Virus (HIV)-negative, not taking medications for seizures and/or thyroid disorders and living in homes built before 1978 (intended to target children at an increased risk of lead exposure). Exclusion criteria included women having a diagnosis of diabetes, bipolar disorder, schizophrenia, or cancer that resulted in radiation treatment or chemotherapy. The IRB at Cincinnati Children’s Hospital Medical Center and all participating hospitals approved this study. All participants provided their written, informed consent for themselves and their children. Of 468 women who enrolled, 67 dropped out before delivery. Among the 389 live born, singleton infants, our analysis included 276 mother-child pairs after excluding those with missing data on the primary outcome measure of autistic traits (SRS scores) (*n* = 106) covariate information (*n* = 7) (Figure S1).

We harmonized exposure and covariate measures from the two cohorts using established procedures [47,48,49]. All covariate information was harmonized to accommodate slight differences in study specific information (e.g., categories of income). Both studies used the same outcome measure, the Social Responsiveness Scale (SRS), described below. Finally, gestational phthalate exposure for both studies was measured at the same laboratory using the same methods. All subjects within the EARLI and HOME studies gave their informed consent for inclusion before they participated in the study. Both studies were conducted in accordance with the Declaration of Helsinki. The EARLI Study protocol was approved by the Institutional Review Boards of the Kaiser Foundation Research Institute, University of California, Davis, VA Northern California Health Care System Human Research Protection Program, Drexel University, and The Children’s Hospital of Philadelphia (120100673), and the HOME Study protocol was approved by the Ethics Committee of Cincinnati Children’s Hospital Medical Center Institutional Review Board (01-8-5, 2008-0022).

### 2.2. Phthalate Exposure Assessment

Maternal urine samples were collected up to three timepoints during pregnancy in EARLI, and twice in HOME. In EARLI, mothers provided up to two first morning void urine samples during their 1st, 2nd, or 3rd trimester. All women in EARLI provided urine during the first trimester, though more women (*n* = 81) provided their second urine sample during the second trimester, compared to the third trimester (*n* = 59). In HOME, urine samples were collected twice, at approximately 16- and 26-week gestation. For both studies, urine samples were stored at ≤−20 °C and sent to the Centers for Disease Control and Prevention (CDC) on dry ice. Laboratory technicians used a modified method of on-line solid phase extraction coupled with isotope dilution-high performance liquid chromatography with tandem mass spectrometry to quantify urinary phthalate metabolite concentrations [50]. Each analytic batch includes reagent blanks and low- and high-concentration quality control (QC) materials, which are evaluated using standard statistical probability rules. The CDC laboratory is licensed by the Clinical Laboratory Improvement Act (CLIA) of 1988. Analytical measurements are conducted following strict quality assurance / quality control (QA/QC) guidelines, CLIA guidelines, and frequent proficiency testing.

We measured maternal urinary concentrations of 9 phthalate metabolites: mono-n-butyl phthalate (MBP), monobenzyl phthalate (MBzP), mono(3-carboxypropyl) phthalate (MCPP), monoethyl phthalate (MEP), mono-isobutyl phthalate (MIBP), and four metabolites of DEHP: mono(2-ethylhexyl) phthalate (MEHP), mono(2-ethyl-5-hydroxyhexyl) phthalate (MEHHP), mono(2-ethyl-5-oxohexyl) phthalate (MEOHP), and mono(2-ethyl-5-carboxypentyl) phthalate (MECPP). To calculate concentrations of ΣDEHP (ng/mL), we divided each DEHP metabolite concentration by its respective molar mass. We then summed the molar concentrations and multiplied them by the molar sum of the molar mass of MECPP. The limits of detection (LODs) for the phthalate metabolites ranged from ~0.2 to ~1 ng/mL. Values below the LOD were assigned values of the LOD/√2 [51].

To control for individual variation in urine dilution, we standardized phthalate metabolite concentrations (ng/mL) by urinary creatinine concentrations (μg/g creatinine). As urinary phthalate concentrations were right skewed, we log_10_-transformed phthalate concentrations to reduce the influence of extreme observations [52]. Among women with more than one urine sample during pregnancy (EARLI: 100% and HOME: 99%), we took the mean of available log_10_-transformed, creatinine-standardized urinary phthalate metabolite concentrations. In cases where we did not have repeated phthalate measures (*n* = 2) we used the one measure available to represent the mean.

### 2.3. ASD-Related Behavior Assessment

Children’s social and communication skills were assessed with the Social Responsiveness Scale (SRS), a validated and reliable questionnaire that assesses the presence and severity of ASD-related behaviors in both general population and clinical settings [53,54,55,56,57,58,59]. The SRS questionnaire includes 65 Likert-style items that measure a continuum of traits exhibited by individuals with ASD, including communication and interpersonal and repetitive/stereotypical behaviors, with subscales for awareness, cognition, communication, mannerisms, and motivation [53,60]. SRS scores are presented as sex-standardized T-scores (mean: 50, standard deviation (SD): 10), where higher scores indicate more ASD-related behaviors. SRS T-scores ranging from 60–75 are indicative of clinically significant deficiencies in reciprocal social behavior that may interfere with daily social interactions, while scores greater than 75 are strongly associated with clinical diagnosis of ASD [58].

Caregivers completed the SRS when children were ages 3 years (SD: 0.13) in EARLI and age 4–8 years (mean: 5.12, SD: 1.74) in HOME. Among HOME Study participants with repeated SRS measures, we used the earliest measurements to align most closely to measures collected in EARLI. Sixty-six percent of HOME Study participants completed the SRS at the age 4 visit (mean: 4.1 years, SD: 0.12), 16% at the age 5 visit (mean: 5.3 years, SD: 0.37), 18% at the age 8 visit (mean: 8.6 years, SD: 0.74). We used SRS T-scores for our primary analysis. Our prior results in HOME showed excellent reproducibility of repeated SRS total T-scores from ages 4–8 years (Intraclass Correlation Coefficient (ICC) = 0.74) [61].

### 2.4. Covariates

We adjusted for sociodemographic and perinatal factors based on biologic plausibility and a priori knowledge using a directed acyclic graph (DAG; Figures S2 and S3). Maternal sociodemographic information and reproductive health information, including race/ethnicity, age, household income, education, and parity, were collected via questionnaires administered by trained research staff. We assessed smoking during pregnancy via cotinine concentrations from urine (measured by immunoassays) in EARLI, and serum (measured by analytic chemistry methods) in HOME. Cotinine concentrations were used as continuous variables in cohort specific models. Previously established thresholds to determine active versus non-active smoking in urine (50 ng/mL) and serum (3 ng/mL) were used in pooled models [62,63].

### 2.5. Statistical Analysis

First, we calculated univariate statistics and characterized distributions of study sample characteristics including maternal sociodemographic and reproductive factors, as well as maternal urinary phthalate metabolite concentrations. Then, we calculated Pearson correlation coefficients between phthalate metabolites and assessed measures of reproducibility of repeated phthalate concentrations using ICCs. Next, we calculated median maternal urinary phthalate concentrations and mean SRS T-scores according to covariates.

Our primary analysis used quantile regression to estimate the unadjusted and covariate-adjusted associations of each log_10_-transformed gestational phthalate metabolite concentration with SRS scores across percentiles of SRS T-scores (i.e., a separate model for each phthalate metabolite) [62,63]. In cohort specific analyses, we adjusted for maternal age, race, parity, cotinine concentrations, and household income. We also adjusted for cohort in pooled analyses.

As an approach, quantile regression is optimal for detecting differences across distributions in cases of skewed outcome data, or if the tails of distributions are suspected of being differentially affected relative to the mean [42]. This technique models associations between exposures and outcome at different percentiles of outcome distributions, and is more robust in capturing associations across the full spectrum of a distribution, especially in regards to extreme values [42,43,44].

Let random variable Y represent the distribution of SRS T-scores which is characterized by its quantile function for any 0<τ<1 as Q(τ)=inf{y:F(y)≥ τ}. Let $y_{i}$ represent the observed SRS T-scores, and $x_{i}$ represent an individual mother’s urinary phthalate concentration. Unlike linear regression, which estimates associations at the mean, quantile regression estimates associations at percentiles of a distribution, indicated as τ. Instead of using least squares to estimate $\beta_{j}$ as in linear regression, at each quantile level τ, the minimization problem is solved using the check loss function to yield a distinct set of regression coefficients:

(1) $$Q_{\tau }\left(y_{i}\right)=\beta_{0}\left(\tau \right)+\;\beta_{1}\left(\tau \right)x_{i1}+\cdots +\;\beta_{p}\left(\tau \right)x_{ip}\;\;\;\;\;\;\;i=1,\ldots ,n$$

We estimated confidence intervals using a rank-based approach. We present results at the 5th, 10th, 25th, 50th, 75th, 90th, and 95th percentiles (where the 5th percentile corresponds to τ=0.05), but show additional quantiles in some cases for illustrative purposes.

We conducted several secondary analyses. First, because ASD is more prevalent in male children than female children [64], we examined whether sex modified the observed associations. Second, given our primary hypothesis that quantile regression may be more informative in modeling the true relations between gestational phthalate exposure and SRS T-scores than linear regression, we also conducted phthalate-specific analyses using linear regression models. In addition to cohort specific analyses, we also conducted analyses using the pooled cohort (EARLI and HOME combined). Finally, we conducted all primary analyses of gestational urinary phthalate metabolite concentrations using raw SRS scores, where we further adjusted for child sex and age.

We conducted several sensitivity analyses to assess the robustness of our results to various adjustments. First, in addition to previously specified covariates, we adjusted for child sex, child age, and pre-pregnancy body mass index (BMI). Second, we jointly adjusted for mono-n-butyl and monobenzyl phthalate concentrations in the same model given their high correlation and common parent metabolite in the case of butylbenzyl phthalate. We also adjusted for neonatal intensive care unit (NICU) admittance, which was available only for a subset of participants in HOME.

We completed statistical analyses using R Studio (version 4.0.3; R Development Core Team), and used the package quantreg for quantile regression analyses [65].

## 3. Results

Women in the EARLI Study were generally older, had higher income, and higher pre-pregnancy BMI compared to women in the HOME Study (Table 1). Both cohorts were predominately non-Hispanic White, college educated, and did not smoke during pregnancy. About half of the women in the HOME Study were nulliparous, whereas all women in EARLI were multiparous by study design. Distributions of study sample characteristics of the two analytic samples did not differ substantially from those of the larger cohorts of enrolled women who delivered live, singleton births, from which they were selected (Table S1).

**Figure 1:**
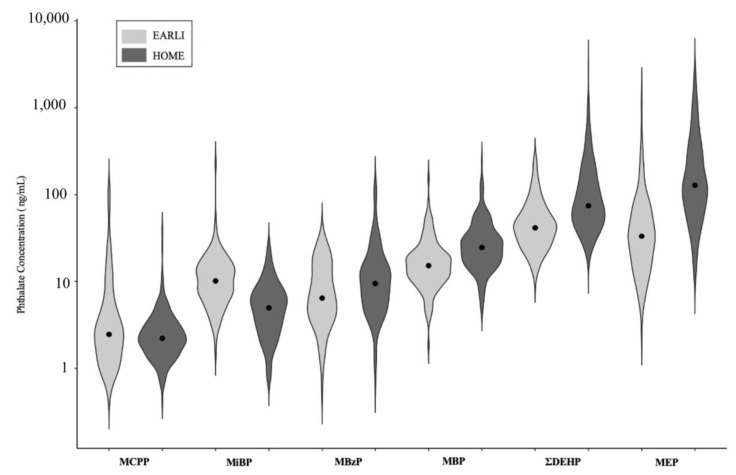
Violin plots of average urinary phthalate metabolite concentrations (ng/mL) during gestation: The Early Autism Risk Longitudinal Investigation (EARLI) (2009–2012) and Health Outcomes and Measures of the Environment (HOME) Studies (2003–2006).

In the HOME Study, women had higher median and maximum concentrations for four of the six gestational urinary phthalate metabolites as compared with women in EARLI (Figure 1, Table S3). Pearson correlation coefficients amongst urinary phthalate metabolite concentrations were generally weakly to moderately positive, with similar correlation patterns in both cohorts (Table S4). Repeated urinary phthalate concentrations had poor to fair reproducibility in both cohorts, although ICCs were slightly higher in EARLI compared with HOME (Table S5) [66]. In both cohorts, ICC values were poor (<0.4) for mono(3-carboxypropyl) phthalate (MCPP), di(2-ethylhexyl) phthalate (ΣDEHP) metabolites, and mono-n-butyl phthalate (MBP) (HOME only), while they were fair (0.4–0.75) for mono-isobutyl phthalate (MiBP), monobenzyl phthalate (MBzP), and monoethyl phthalate (MEP).

Dot in each violin plot represents the median urinary phthalate metabolite concentration. Shaded areas represent the density function of urinary phthalate metabolite concentration. Lighter colors indicate distributions in EARLI while darker colors represent HOME. Values correspond to average maternal metabolite concentrations among all participants in EARLI (*n* = 140) and HOME (*n* = 276). MCPP, mono(3-carboxypropyl) phthalate; MiBP, mono-isobutyl phthalate; MBzP, monobenzyl phthalate; MBP, mono-n-butyl phthalate; ΣDEHP, summary di(2-ethylhexyl) phthalate metabolite measure; MEP, monoethyl phthalate. Concentrations of ΣDEHP (in ng/mL) were calculated using the following formula: ΣDEHP (ng/mL) = [MECPP (ng/mL)/278 g/mol + MEHHP (ng/mL)/294.3 g/mol + MEOHP (ng/mL)/292.2 g/mol + MEHP (ng/mL)/278.3 g/mol] × 278 g/mol [67]. Phthalate metabolites are ordered based on median concentration values from left (lowest median concentrations) to right (highest median concentrations).

Bivariate relations between some covariates and median urinary concentrations of phthalate metabolites, varied between the two cohorts, particularly maternal race, age, parity, and pre-pregnancy BMI. In both cohorts, however, urinary phthalate concentrations were higher in smokers than non-smokers. In HOME, phthalate metabolites were generally higher among those who identified as Non-White, were multiparous, and overweight or obese prior to pregnancy. In HOME, maternal urinary concentrations for phthalate metabolites were inversely associated with maternal age, maternal educational attainment, and annual income (Table S2).

The distributions of SRS T-scores were slightly lower in EARLI (mean: 48, SD: 11) compared to HOME (mean: 52, SD: 10). In contrast, the proportion of children with SRS scores ≥60 and ≥75 were similar in EARLI and HOME (Figure S4). In both cohorts, SRS T-scores were inversely associated with maternal age, education, and income, and positively associated with smoking and pre-pregnancy BMI. Higher SRS T-scores were associated with Non-White maternal race in HOME, but not in EARLI (Table 1).

### 3.1. Gestational Maternal Urinary Phthalate Concentrations and Social Responsiveness Scores

**Figure 2:**
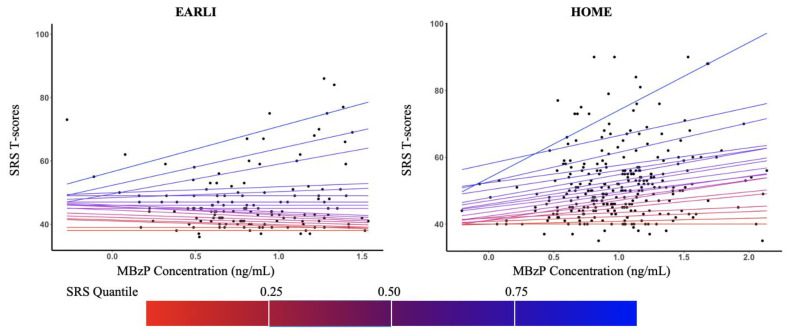
Adjusted differences in children’s SRS T-scores at Ages 3 to 8 per 10-fold increase in gestational urinary monobenzyl phthalate (MBzP) concentration from 0.05–0.95 (at intervals of 0.05) quantiles of SRS T-scores: the EARLI (2009–2012) and HOME Studies (2003–2006). EARLI: Early Autism Risk Longitudinal Investigation Study, HOME: Health Outcomes and Measures of the Environment Study, SRS: Social Responsiveness Scale, mono-isobutyl phthalate; MBzP. Adjusted for maternal age (continuous), maternal race (white vs. non-white), income (<$30,000 vs. $30,000–$75,000, ≥$75,000), parity (continuous), and log10-transformed urine/serum cotinine concentrations (continuous). Note cotinine concentrations were measured in maternal urine in EARLI and serum in HOME. Positive slope for SRS indicate that maternal phthalate exposure is associated with more deficits in social responsiveness traits. Quantile sequence 0.05–0.95 by 0.05.

**Figure 3:**
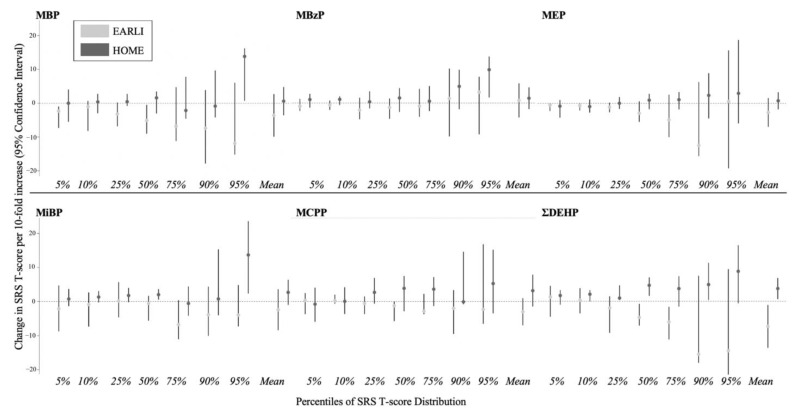
Adjusted differences in child SRS T-scores at 3 to 8 years per 10-fold increase in gestational maternal urinary phthalate metabolite concentrations: the EARLI (2009–2012) and HOME Studies (2003–2006). EARLI: Early Autism Risk Longitudinal Investigation Study, HOME: Health Outcomes and Measures of the Environment Study, SRS: Social Responsiveness Scale. MCPP, mono(3-carboxypropyl) phthalate; MiBP, mono-isobutyl phthalate; MBzP, monobenzyl phthalate; MBP, mono-n-butyl phthalate; ΣDEHP, summary di(2-ethylhexyl) phthalate metabolite measure; MEP, monoethyl phthalate. Concentrations of ΣDEHP (in ng/mL) were calculated using the following formula: ΣDEHP (ng/mL) = [MECPP (ng/mL)/278 g/mol + MEHHP (ng/mL)/294.3 g/mol + MEOHP (ng/mL)/292.2 g/mol + MEHP (ng/mL)/278.3 g/mol] × 278 g/mol. Adjusted for maternal age (continuous), maternal race (white vs. non-white), income (<$30,000 vs. $30,000–$75,000, ≥$75,000), parity (continuous), and log10-transformed urine/serum cotinine concentrations (continuous). Note cotinine concentrations were measured in maternal urine in EARLI and serum in HOME. Positive coefficients for SRS indicate that maternal phthalate exposure is associated with more deficits in social responsiveness traits. Mean values represent effect measures from linear regression analyses. Note that values may appear to differ slightly from those reported in Table S6, as we used un-rounded values to generate these figures. Phthalate metabolites are ordered based on median concentration values from left (lowest median concentrations) to right (highest median concentrations).

For most phthalate metabolites, adjusted differences in SRS T-scores varied in magnitude and direction across percentiles of SRS T-scores (5th, 10th, 25th, 50th, 75th, 90th, and 95th), and by cohort (Figure 2). Several urinary phthalate metabolites were associated with children’s SRS T-scores, particularly at higher percentiles of SRS T-scores. In both cohorts, we observed increasingly stronger, positive associations of MBzP concentrations with child SRS T-Scores from the 50th to 95th percentiles of the SRS-T scores, though effect sizes were much larger in HOME (EARLI: 50th [β: −1; 95% confidence interval (CI): −5, 1], 95th [β: 3; 95% CI: −9, 8]; HOME: 50th [β: −1; 95% CI: −3, 4], 95th [β: 10; 95% CI: 2, 14] (Figure 3)). With the exception of MBzP, the pattern of associations between urinary phthalate metabolite concentrations and SRS T-scores varied between EARLI and HOME (Figure S5). In EARLI, associations for phthalate metabolites MBP, MEP, and ΣDEHP were generally negative, and were null for phthalate metabolites MIBP and MCPP across SRS T-Score percentiles.

In HOME, we observed increasingly positive associations of MBP, MBzP, MiBP, MCPP, and ΣDEHP with SRS T-scores across increasing percentiles of SRS T-scores. Associations between urinary phthalate concentrations and SRS-scores were consistently stronger at the 95th percentile compared to all other percentiles in HOME. Associations between MEP concentrations and SRS-T scores monotonically increased from the 75th to 95th percentile rising from approximately −1 to 1 at the 5th to 75th percentile to 2 (95% CI: −4, 9) at the 90th and to 3 (95% CI: −6, 19) at the 95th percentile (Table S5).

### 3.2. Secondary and Sensitivity Analyses

Generally, the results from adjusted linear regression models suggested inverse and imprecise associations between phthalate metabolite concentrations and SRS T-scores in EARLI. In HOME, associations were positive; however, the magnitude of associations was modest (e.g., MEP: β: 1; 95% CI: −2, 3; ΣDEHP: β: 4; 95% CI: 1, 7). As expected, linear regression results were comparable to quantile regression results obtained at the 50th percentile of SRS T-scores (Table S7).

**Figure 4:**
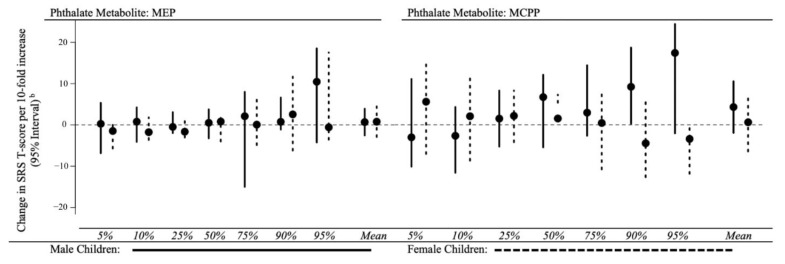
Adjusted differences in child SRS T-scores at 3 to 8 years per 10-fold increase in gestational maternal urinary MEP and MCPP concentrations, stratified by child sex: HOME study (2003–2006). HOME: Health Outcomes and Measures of the Environment Study, SRS: Social Responsiveness Scale, MEP: monoethyl phthalate; MCPP: mono(3-carboxypropyl) phthalate. Adjusted for maternal age (continuous), maternal race (white vs. non-white), income (<$30,000 vs. $30,000–$75,000, ≥$75,000), parity (continuous), and log10-transformed urine/serum cotinine concentrations (continuous). Note cotinine concentrations were ascertained from maternal urine in HOME. Positive coefficients for SRS indicate that maternal phthalate exposure is associated with more deficits in social responsiveness traits. Note that values may appear to differ slightly from those reported in Table S8, as we used un-rounded values to generate these figures.

In EARLI, we did not observe evidence for effect measure modification when we stratified by child sex for phthalate metabolites MBzP, MiBP, MCPP, or ΣDEHP. While associations between urinary MBP and MEP varied by child sex at the 90th and 95th percentiles of SRS T-scores, 95% confidence intervals were large and imprecise (Table S8). Associations between gestational urinary metabolites MBP, MBzP, MEP, and ΣDEHP in HOME were similar between boys and girls at lower percentiles (≤75th); however, at the highest percentiles we observed a larger magnitude of association in boys than girls. There were increasing positive associations between MCPP concentrations and SRS T-scores across increasing percentiles of SRS T-scores for boys, while associations were null in girls (Figure 4).

When conducting analyses using SRS raw scores, instead of T-scores, we did not observe any substantial changes in patterns of results. This was consistent amongst all urinary phthalate metabolites in both cohorts (Table S9).

Adjusting for child sex or child age did not substantially change our results for MBzP in either cohort. Adjustment for pre-pregnancy BMI attenuated associations for EARLI at the 90th (β: 0; 95% CI: −10, 10 vs. β: 1; 95% CI: -10, 10) and 95th (β: 0; 95% CI: −7, 7 vs. β: 3; 95% CI: −9, 8) percentiles, and the 95th (β: 7; 95% CI: 6, 15 vs. β: 10; 95% CI: 2, 14) percentile in HOME. Additional adjustment for MBP did not substantially change our results within HOME. However, in EARLI additional adjustment for MBP increased the magnitude of the association, particularly at the 75th (β: 2; 95% CI: -3, 8 vs. β: -1; 95% CI: −4, 4) and 95th (β: 13; 95% CI: −7, 22 vs. β: 3; 95% CI: −9, 8) SRS T-score percentiles. Additional adjustment for NICU admittance did not substantially change out results within HOME (Table S10).

## 4. Discussion

Guided by the hypothesis that impacts of environmental insults may differ at the ‘tails’ of neurodevelopmental trait distributions due to differences in susceptibility, we assessed the relation between maternal urinary phthalate concentrations during pregnancy and behavior traits related to ASD in children using quantile regression. In HOME, a general population cohort, we observed stronger positive associations of urinary phthalate MBP, MBzP, MiBP, MCPP, and ΣDEHP concentrations with parent-reported ASD-related behaviors at higher percentiles (≥75%) of child SRS T-scores, relative to lower ones. This may suggest the effects of phthalates varies as a function of this propensity towards ASD-related traits (e.g., higher SRS T-scores). In EARLI, the enriched risk cohort of mothers who previously had a child with ASD, we found inverse or null associations, with the exception of MBzP, between gestational urinary phthalate concentrations and SRS T-scores.

Previous studies suggest that gestational urinary phthalate concentrations are adversely associated with multiple long-term child neurodevelopmental outcomes [68]. This includes deficits in cognitive abilities [37,69] and motor development [70], as well as increased risk of internalizing and externalizing behaviors [71,72,73], and Attention Deficit Hyperactivity Disorder (ADHD) [74]. Some studies report sex-specific effects [14,37,38,39,40,75,76,77].

Some studies suggest modest increases in ASD related traits with higher gestational phthalate metabolite concentrations [34,36,41], while others report null findings [37,38,39]. In prior studies, increasing concentrations of phthalate metabolites MBP, MCPP, MEP, and summary measures of low-molecular weight phthalates (including MBP, MEP, MMP, and MiBP) were found to have the strongest associations with increases in SRS T-scores [34,37,41]. We too observed stronger associations for MBP, MiBP, MCPP in HOME. When we examined these associations using linear regression models in HOME, we observed modest (MBzP, MiBP, MCPP, and ΣDEHP), or null associations (MBP, MEP) with child SRS T-scores, which was consistent with some prior literature [34,35,40]. Only when we assessed this relationship across percentiles of child SRS T-scores, did we observe associations of gestational MBP, MiBP, MCPP, MBzP, and ΣDEHP concentrations with SRS T-Scores in HOME. One potential reason for the discrepancy between our results and prior studies is that prior studies estimated associations at the mean of the outcome [34,35,36,37,38,39,40], and these individuals at the mean may be less susceptible compared to those at higher percentiles of SRS scores.

Phthalates have been found to reduce fetal androgen production, which may influence neurodevelopmental processes differently in boys and girls [78]. Thus, boys may be more ‘susceptible’ at the upper tail of the SRS T-score distribution, relative to boys at lower percentiles or girls at the same percentile given the potential role of testosterone in the development of ASD and related traits [79,80,81]. The anti-androgenic effects of some phthalate metabolites may contribute to the sex-specific associations observed in prior literature, as well as the findings we observed in HOME [26,27,28,29,30,82,83].

We observed larger effect sizes in HOME relative to EARLI, where associations were largely null, or inverse. Results were robust to adjustment by child sex and pre-pregnancy BMI in sensitivity analyses. A previous study using a different ASD enriched risk cohort also reported inverse or null associations between maternal urinary phthalate concentrations and child SRS T-scores [39]. Several reasons could account for the discrepancies in findings between HOME and EARLI. The EARLI and HOME studies also vary in terms of design and participant recruitment. In EARLI, the enriched risk cohort study was designed with the intention of exploring genetic effects of ASD. The null associations in EARLI could be a function of the strong genetic components to ASD, which may mask associations between environmental exposures and child neurodevelopmental outcomes [39]. It is also possible that EARLI study participants may have been more motivated to reduce their exposure to known and suspected neurotoxicants, given that they already had a child with ASD and thus may be aware of potential risk factors. However, this seems unlikely for phthalates given the similarity in the median and range of urinary phthalate metabolite concentrations in the two cohorts.

HOME study participants generally had modestly higher median values and larger ranges of phthalate metabolites compared to EARLI. Distributions of demographic and reproductive characteristics differed between these cohorts, as well as patterns of measured phthalate concentrations by study sample characteristics. Given the differences in design and source populations between EARLI and HOME, it is possible that the patterns of use for phthalate containing products were also differential by cohort. It is also possible that missing data could induce selection bias within the analytic sample, and this is a potential limitation that should be addressed in future studies.

This study has several strengths and limitations. First, gestational phthalate exposure may be misclassified due to the poor to fair within-person variability of urinary phthalate metabolite concentrations. While phthalate have short half-lives, and are rapidly excreted from the body [11,12,13], we reduced some of this measurement error given that all EARLI and most (>98%) HOME study participants provided two urine samples. Future work would benefit from collecting more urinary biomarkers to enhance exposure assessment, as well as investigating periods of susceptibility throughout the gestational period. Additionally, assessing urinary phthalate metabolite concentrations in first morning voids may also reduce exposure measurement error by providing more accurate predictions of average daily metabolite concentrations, though measures could be biased against certain exposures (e.g., personal care products) that are only used in morning [84,85].

Second, quantile regression has some advantages and limitations. The primary advantage of this approach is the ability to estimate associations across percentiles of the outcome, emphasizing trends in magnitude of the exposure-outcome association across the distribution of the outcome rather than at the mean of the outcome. This highlights important theoretical implications for health promotion among all children, as methods to evaluate the impact of chemical exposures on neurodevelopmental traits for the average child may not be applicable to those who are most vulnerable to environmental exposures. Additionally, the effect estimates from quantile regression are less influenced by outliers and quantile regression does not require the same distributional assumptions as other regression methods [42,43,44]. Limitations include reduced power for analyses in sub-populations across percentiles of the outcome distribution [42]. Additionally, coefficients and confidence intervals corresponding to estimates for higher percentiles of the SRS T-score distribution should be interpreted with caution, as smaller sample sizes can contribute to the imprecise and asymmetric confidence intervals [42,43,44]. We did not assess the cumulative effects of exposure to multiple phthalates, but weighted quantile sum regression, Bayesian kernel machine regression, and other methods should be considered in future studies [86,87].

Third, while we adjusted for many potential confounding variables in both primary and secondary analyses, residual confounding is still a possibility. For example, given limitations in data availability in the EARLI and HOME studies, we were unable to account for sources of phthalate exposure by considering frequency and quantity of personal care product use. We were also unable to account for other maternal dietary factors such as fast food consumption [15,88] (associated with exposure to some phthalate metabolites), or fish oil supplementation (potentially associated with improved neurodevelopmental outcomes and reduced risk of ASD) [89].

## 5. Conclusions

In two prospective cohorts, we investigated if sub-populations at the ‘tails’ of ASD-related traits were more susceptible to the potential effects of phthalate exposure using quantile regression. In HOME, but not EARLI, we saw evidence of a positive association between most gestational urinary phthalates with SRS T-scores at higher percentiles of SRS T-scores. This suggests children who exhibit more ASD related traits may represent a vulnerable subgroup of children who may be particularly susceptible to the neurotoxic effects of phthalates during gestation. We speculate that differences in findings between these cohorts could be due to variations in the underlying genetic susceptibility, which might be greater than environmental effects in this cohort, as well as differences in phthalate metabolite concentrations, covariates, and exposure misclassification. Future studies should consider the use of quantile regression as a valuable tool to explore patterns of associations across outcome distributions.

## Figures and Tables

**Table 1:** Study sample characteristics and mean child Social Responsiveness Scale (SRS) total T-Scores at 3 to 8 years according to covariates: the EARLI (2009–2012) and HOME Studies (2003–2006).

Variable			SRS T-Scores	
	EARLI	HOME	EARLI	HOME
	N (%)	N (%)	Mean (SD)	Mean (SD)
Overall	140 (100)	276 (100)	48 (11)	52 (10)
Maternal Age				
<25 years	2 (1)	59 (21)	48 (1.4)	57 (12)
25–<35 years	68 (49)	170 (62)	50 (13)	50 (8.1)
35+ years	70 (50)	47 (17)	46 (7.6)	52 (13)
Maternal Race				
White	95 (68)	178 (64)	48 (10)	49 (8.5)
Non-White	45 (32)	98 (36)	48 (12)	57 (12)
Maternal Education				
High School or less	14 (10)	64 (23)	50 (10)	58 (12)
Some College	40 (29)	75 (27)	53 (15)	53 (9.4)
Completed College	86 (61)	137 (50)	45 (7.5)	48 (8.3)
Annual Income				
<$30,000	15 (11)	87 (32)	53 (17)	58 (12)
$30,000–$75,000	42 (30)	87 (32)	50 (12)	50 (8.9)
≥$75,000	83 (59)	102 (36)	46 (7.9)	47 (7.4)
Maternal Smoking ^1,2^				
Non-Smoking	132 (94)	245 (89)	47 (10)	52 (11)
Active Smoking	8 (6)	31 (11)	60 (19)	55 (8.7)
Parity ^3^				
0	--	128 (46)	--	51 (10)
1	66 (47)	86 (31)	49 (12)	51 (10)
2+	74 (53)	62 (23)	47 (10)	55 (11)
Pre-pregnancy BMI (kg/m^2^)				
Normal/Underweight <25	54 (39)	142 (51)	45 (8.5)	50 (11)
Overweight ≥25–<30	40 (29)	69 (25)	47 (9.0)	51 (8.2)
Obese ≥30	46 (33)	65 (24)	52 (13)	55 (12)
Child Sex				
Male	77 (55)	123 (45)	50 (13)	51 (9.7)
Female	63 (45)	153 (55)	45 (6.9)	53 (11)

BMI: body mass index, EARLI: Early Autism Risk Longitudinal Investigation Study, HOME: Health Outcomes and Measures of the Environment Study, SRS: Social Responsiveness Scale. ^1^ Maternal smoking during pregnancy for EARLI was based on maternal urinary cotinine concentrations (a metabolite of nicotine) during pregnancy. The cut off point of 50 ng/mL was used to differentiate between non-smoking and active smoking [62] ^2^ Maternal smoking during pregnancy for HOME estimated based on maternal serum cotinine concentrations during pregnancy. The cut off point of 3.0 ng/mL was used to differentiate non-smoking and active smoking [61]. ^3^ Note that for parity, the EARLI cohort consists of mothers who had at least one previous child.

## Data Availability

There are ethical restrictions on sharing the de-identified data. Reasons include that the data contains potentially identifiable information. However, the data is available upon request. The EARLI and HOME Study principal investigators have actively engaged in collaborative data-sharing projects. We welcome new collaborations with other investigators. Interested investigators in EARLI and HOME study data should contact Katelyn Lutz (katelyn_lutz@cchmc.org) to discuss collaborative opportunities, obtain additional information about the EARLI and HOME Studies and request a project proposal form. The Data Sharing Committees of both studies meet regularly to review proposed research projects and ensure that they do not overlap with extant projects and are an efficient use of scarce resources (e.g., cord blood).

## Supplementary Materials

The following are available online at https://www.mdpi.com/1660-4601/18/3/1254/s1, Table S1: Study Sample Characteristics and Mean Child SRS Total T-Scores at 3 to 8 Years According to Covariates in Full Cohort Samples Compared to Analytic Sample: The EARLI (2009–2012) and HOME Studies (2003–2006), Table S2: Study Sample Characteristics and Median Maternal Urinary Phthalate Concentrations (ng/mL) According to Covariates: The EARLI (2009–2012) and HOME Studies (2003–2006), Table S3: Univariate statistics of repeated Maternal Urinary Concentrations of Phthalate Metabolites (ng/mL): The EARLI (2009–2012) and HOME Studies (2003–2006), Table S4: Pearson correlation coefficients between log-10 transformed gestational urinary phthalate metabolites (ng/mL): The EARLI (2009–2012) and HOME Studies (2003–2006), Table S5: Intra-class correlation coefficients and (95% Confidence Intervals (CI)) for gestational urinary phthalate metabolites (ng/mL): The EARLI (2009–2012) and HOME Studies (2003–2006), Table S6: Unadjusted and adjusted differences (95% Confidence Intervals (CI)) in Child SRS T-Scores at 3 to 8 Years Per 1-Unit Increase Per 10-fold Increase in Gestational Urinary Phthalate Metabolite Concentrations at Quantiles of SRS T-Scores: The EARLI (2009–2012) and HOME Studies (2003–2006). Table S7: Unadjusted and Adjusted Differences in Children’s SRS T-Scores at Ages 3 to 8 Per 1-Unit Increase Per 10-fold Increase in Gestational Urinary Phthalate Metabolite Concentrations Using Linear Regression: The EARLI (2009–2012) and HOME Studies (2003–2006), Table S8: Unadjusted and Adjusted Differences in Children’s SRS T-Scores at Ages 3 to 8 Per 1-Unit Increase Per 10-fold Increase in Gestational Urinary Phthalate Metabolite Concentrations Using Quantile Regression, Stratified by Child Sex: The EARLI (2009–2012) and HOME Studies (2003–2006), Table S9: Adjusted differences (95% Confidence Intervals (CI)) in Child SRS T-Scores at 3 to 8 Years Per 1-Unit Increase Per 10-fold Increase in Gestational Urinary Monobenzyl Phthalate (MBzP) Metabolite Concentrations at Quantiles of SRS T-Scores: The EARLI (2009–2012) and HOME Studies (2003–2006), Table S10: Adjusted differences (95% cConfidence iIntervals (CI)) in cChild SRS T-sScores at 3 to 8 yYears pPer 10-fold iIncrease in gGestational uUrinary mMonobenzyl pPhthalate (MBzP) mMetabolite cConcentrations at qQuantiles of SRS T-sScores c: tThe EARLI (2009–2012) and HOME Studies (2003–2006). Figure S1: Flow Chart of Participant Selection to Final Sample Size, Figure S2: Directed Acyclic Graph used to Select Covariates for our Primary Analysis in the Association Between Maternal Urinary Phthalate Concentrations During Gestation and Child SRS Scores, Figure S3: Directed Acyclic Graph used to Select Covariates for our Primary and Secondary Analysis in the Association Between Maternal Urinary Phthalate Concentrations During Gestation and Child SRS Scores, Figure S4: Kernel density plot of distributions of Children’s SRS T-Score at Ages 3 The EARLI (2009–2012) and HOME Studies (2003–2006), Figure S5: Adjusted Differences in Children’s SRS T-Scores at Ages 3 to 8 Per 1-Unit Increase Per 10-fold Increase In Gestational Urinary Phthalate Metabolite Concentrations Using Quantile Regression: The EARLI (2009–2012) and HOME Studies (2003–2006).
