# Supplementary material for: Gestational Exposure to Phthalates and Social Responsiveness Scores in Children Using Quantile Regression: The EARLI and HOME Studies
Source: Int J Environ Res Public Health. 2021 Jan 30;18(3):1254. doi: 10.3390/ijerph18031254 (PMC7908417; doi:10.3390/ijerph18031254)
Supplement: Supplementary file 1 [file ijerph-18-01254-s001.pdf]

Supplemental Material

Gestational Exposure to Phthalates and Social Responsiveness Scores in Children Using Quantile Regression: The EARLI and HOME Studies

Marisa A. Patti, Craig Newschaffer, Melissa Eliot, Ghassan B. Hamra, Aimin Chen, Lisa A Croen, Margaret Daniele Fallin, Irva Hertz-Picciotto, Geetika Kalloo, Jane Khoury, Bruce P. Lanphear, Kristen Lyall, Kimberly Yolton, and Joseph M. Braun

CONTENTS

**Table S1**. Study Sample Characteristics and Mean Child SRS Total T-Scores According to Covariates in Full Cohort Samples Compared to Analytic Sample……………………………….	2
**Table S2**. Study Sample Characteristics and Median Maternal Urinary Phthalate Concentrations According to Covariates…………………………………………………….	3
**Table S3**. Univariate statistics of repeated Maternal Urinary Concentrations of Phthalate Metabolites…………………………………………………………………………………...	5
**Table S4**. Pearson correlation coefficients between gestational urinary phthalate metabolites….	6
**Table S5**. Intra-class correlation coefficients for gestational urinary phthalate metabolites……..	7
**Table 6**: Unadjusted and adjusted differences in Child SRS T-Scores Per 10-fold Increase in Gestational Urinary Phthalate Metabolite Concentrations at Quantiles of SRS T-Scores………..	8
**Table S7**: Unadjusted and Adjusted Differences in Children's SRS T-Scores Per 10-fold Increase in Gestational Urinary Phthalate Metabolite Concentrations Using Linear Regression...	10
**Table S8**. Unadjusted and Adjusted Differences in Children's SRS T-Scores Per 10-fold Increase in Gestational Urinary Phthalate Metabolite Concentrations Using Quantile Regression, Stratified by Child Sex…………………………………………………………….	11
**Table S9.** Unadjusted and adjusted differences in Child SRS raw scores at 3 to 8 Years Per 10-fold Increase in Gestational Urinary Phthalate Metabolite Concentrations at Quantiles of SRS Raw Scores…………………………………………………………………………….….	13
**Table S10**: Adjusted differences in Child SRS T-Scores Per 10-fold Increase in Gestational Urinary Monobenzyl Phthalate Metabolite Concentrations at Quantiles of SRS T-Scores………	15
**Figure S1.** Flow Chart of Participant Selection to Final Sample Size………………………….	16
**Figure S2.** Directed Acyclic Graph used to Select Covariates for our Primary Analysis in the Association Between Maternal Urinary Phthalate Concentrations During Gestation and Child SRS Scores………………………………………………………………………………..	17
**Figure S3.** Directed Acyclic Graph used to Select Covariates for our Primary and Secondary Analysis in the Association Between Maternal Urinary Phthalate Concentrations During Gestation and Child SRS Scores………………………………………………………..	18
**Figure S4.** Kernel density plot of distributions of Children's SRS T-Score……………………...	19
**Figure S5**. Adjusted Differences in Children's SRS T-Scores Per 10-fold Increase in Gestational Urinary Phthalate Concentrations Using Quantile Regression……………………….	20

Table S1. Study sample characteristics and mean child SRS total T-scores at 3 to 8 years according to covariates in full [a] cohort samples compared to analytic sample: the EARLI (2009–2012) and HOME Studies (2003–2006).

	EARLI				HOME			
			SRS T-Scores				SRS T-Scores	
Variable	Analytic Sample	Full Sample	Analytic Sample	Full Sample	Analytic Sample	Full Sample	Analytic Sample	Full Sample
	N (%)		Mean (SD)		N (%)		Mean (SD)	
Overall	140 (100)	176 (100)	48 (11)	48 (11)	276 (100)	283 (100)	52 (10)	52 (10)
Maternal Age								
<25 years	2 (1)	8 (5)	48 (1.4)	55 (11)	59 (21)	62 (22)	57 (12)	57 (12)
25 - <35 years	68 (49)	85 (48)	50 (13)	50 (13)	170 (62)	174 (61)	50 (8.1)	50 (8.1)
35+ years	70 (50)	83 (47)	46 (7.6)	46 (8.1)	47 (17)	52 (18)	52 (13)	51 (13)
Maternal Race								
White	95 (68)	110 (63)	48 (10)	48 (11)	178 (64)	183 (65)	49 (8.5)	49 (8.5)
Non-White	45 (32)	66 (38)	48 (12)	49 (11)	98 (36)	100 (35)	57 (12)	56 (12)
Maternal Education								
High School or less	14 (10)	25 (14)	50 (10)	51 (9.3)	64 (23)	65 (23)	58 (12)	58 (12)
Some College	40 (29)	50 (28)	53 (15)	53 (14)	75 (27)	76 (27)	53 (9.4)	53 (9.4)
Completed College	86 (61)	107 (61)	45 (7.5)	46 (8.3)	137 (50)	144 (51)	48 (8.3)	48 (8.2)
Annual Income								
<$30,000	15 (11)	29 (16)	53 (17)	55 (14)	87 (32)	89 (31)	58 (12)	58 (12)
$30,000 - $75,000	42 (30)	50 (28)	50 (12)	49 (12)	87 (32)	90 (32)	50 (8.9)	50 (8.8)
≥$75,000	83 (59)	97 (55)	46 (7.9)	46 (8.7)	102 (36)	104 (37)	47 (7.4)	47 (7.4)
Maternal Smoking [b, c]								
Non-Smoking	132 (94)	148 (84)	47 (10)	48 (9.9)	245 (89)	252 (89)	52 (11)	51 (10)
Active Smoking	8 (6)	8 (5)	60 (19)	60 (19)	31 (11)	31 (11)	55 (8.7)	55 (8.7)
Parity [d]								
0	--	--	--	--	128 (46)	131 (46)	51 (10)	51 (10)
1	66 (47)	84 (48)	49 (12)	49 (12)	86 (31)	93 (33)	51 (10)	51 (10)
2 +	74 (53)	95 (54)	47 (10)	47 (10)	62 (23)	65 (23)	55 (11)	55 (11)
Pre-pregnancy BMI [e]								
Normal/Underweight	54 (39)	73 (41)	45 (8.5)	46 (8.7)	142 (51)	149 (53)	50 (11)	50 (11)
Overweight	40 (29)	56 (32)	47 (9.0)	47 (9.6)	69 (25)	76 (27)	51 (8.2)	51 (8.2)
Obese	46 (33)	61 (35)	52 (13)	52 (13)	65 (24)	72 (25)	55 (12)	55 (12)
Child Sex								
Male	77 (55)	97 (55)	50 (13)	51 (13)	123 (45)	126 (45)	51 (9.7)	51 (9.6)
Female	63 (435)	79 (45)	45 (6.9)	45 (6.8)	153 (55)	157 (55)	53 (11)	53 (11)

BMI: body mass index, EARLI: Early Autism Risk Longitudinal Investigation Study, HOME: Health Outcomes and Measures of the Environment Study, SRS: Social Responsiveness Scale

[a] Note that values may not sum to the full sample size due to missing information from subsets of the original full sample

[b] Maternal smoking during pregnancy for EARLI was based on maternal urinary cotinine concentrations (a metabolite of nicotine) during pregnancy. The cut off point of 50 ng/ml was used to differentiate between non-smoking and active smoking [62].

[c] Maternal smoking during pregnancy for HOME estimated based on maternal serum cotinine concentrations during pregnancy. The cut off point of 3.0 ng/ml was used to differentiate non-smoking and active smoking [61].

[d] Note that for parity, the EARLI cohort consists of mothers who had at least one previous child.

[e] Pre-pregnancy BMI (kg/m2) was defined as normal/underweight <25, overweight ≥25-<30, and obese ≥ 30

Table S2. Study sample characteristics and median maternal urinary phthalate concentrations (ng/mL) according to covariates: the EARLI (2009–2012) and HOME Studies (2003–2006).

Table S1.a EARLI

	Median (25th & 75th percentile)					
Variable	MCPP	MiBP	MBzP	MBP	ΣDEHP	MEP
Overall	2.1 (1.0, 5.6)	9.4 (4.7, 17)	6.0 (3.0, 14)	13 (7.5, 25)	35 (19, 70)	27 (14, 62)
Maternal Age						
<25 years	1.2 (1.1, 2.4)	10 (7.0, 18)	6.4 (5.3, 9.0)	11 (9.7, 12)	21 (20, 182)	16 (12, 18)
25-<35 years	2.2 (1.0, 6.0)	9.8 (4.7, 19)	7.5 (3.6, 17)	15 (8.0, 33)	43 (19, 74)	29 (15, 63)
35+ years	2.1 (1.1, 5.2)	9.2 (4.7, 15)	5.0 (2.8, 9.9)	12 (6.9, 20)	31 (19, 58)	26 (12, 62)
Maternal Race						
White	2.7 (1.3, 7.1)	9.9 (4.5, 18)	6.0 (3.0, 14)	14 (7.5, 28)	36 (21, 70)	24 (13, 59)
Non-White	1.6 (0.7, 3.5)	7.8 (4.9, 16)	6.0 (2.9, 14)	12 (7.5, 20)	33 (19, 70)	32 (17, 85)
Maternal Education						
High School or less	1.9 (0.9, 5.9)	7.3 (4.4, 23)	7.2 (3.6, 15)	14 (6.9, 23)	37 (19, 69)	19 (13, 32)
Some College	2.1 (1.2, 7.0)	9.7 (5.1, 17)	7.4 (3.5, 18)	15 (7.7, 28)	35 (21, 70)	35 (19, 76)
Completed College	2.4 (1.0, 5.2)	9.2 (4.7, 15)	5.1 (2.8, 12)	13 (7.5, 23)	35 (19, 67)	25 (13, 60)
Annual Income						
<$30,000	1.9 (0.9, 5.7)	8.3 (3.4, 17)	8.0 (3.5, 14)	15 (9.0, 20)	47 (19, 81)	29 (15, 63)
$30,000 - $75,000	2.2 (1.2, 5.6)	5.8 (13, 20)	8.8 (4.2, 18)	16 (8.4, 33)	40 (23, 71)	43 (19, 86)
≥$75,000	2.2 (1.0, 5.5)	9.0 (3.9, 15)	4.7 (2.5, 10)	12 (7.2, 20)	31 (19, 62)	23 (12, 47)
Maternal Smoking [a,b]						
Non-Smoking	2.1 (1.0, 5.5)	9.2 (4.7, 17)	5.9 (2.9, 13)	13 (7.5, 23)	35 (19, 69)	25 (14, 62)
Active Smoking	3.1 (0.9, 6.4)	14 (6.4, 17)	13 (11, 19)	19 (12, 33)	34 (17, 70)	47 (14, 77)
Parity [c]						
0	--	--	--	--	--	--
1	2.7 (1.1, 7.2)	9.6 (5.0, 16)	5.9 (2.5, 11)	14 (7.1, 23)	34 (19, 69)	26 (13, 63)
2 +	1.0 (1.0, 4.3)	9.0 (4.2, 17)	6.2 (3.3, 17)	12 (7.8, 26)	36 (21, 70)	27 (14, 62)
Pre-pregnancy BMI (kg/m2)						
Normal/Underweight <25	2.7 (1.3, 7.2)	8.7 (4.9, 16)	5.5 (2.7, 12)	14 (7.4, 20)	36 (19, 83)	29 (14, 69)
Overweight ≥25, <30	1.8 (0.9, 6.1)	9.6 (4.5, 17)	6.4 (3.2, 12)	11 (7.2, 27)	36 (21, 67)	25 (14, 59)
Obese ≥30	2.1 (0.9, 4.0)	9.9 (4.9, 17)	7.2 (3.7, 17)	14 (7.9, 26)	35 (19, 64)	25 (14, 47)
Child Sex						
Male	1.7 (0.9, 4.0)	8.8 (4.1, 16)	5.9 (2.9, 12)	12 (7.3, 21)	30 (19, 56)	24 (13, 62)
Female	2.9 (1.3, 7.1)	10 (5.13, 19)	6.3 (3.1, 17)	15 (8.4, 29)	46 (21, 73)	29 (15, 65)

Table S1.b HOME						
	Median (25th & 75th percentile)					
Variable	MCPP [d]	MiBP	MBzP	MBP	ΣDEHP	MEP
Overall	2.2 (1.1, 4.4)	5.0 (1.6, 11)	9.4 (3.7, 23)	23 (10, 49)	77 (32, 175)	117 (44, 352)
Maternal Age						
<25 years	2.8 (1.4, 5.2)	8.5 (3.6, 15)	19 (8.2, 37)	41 (16, 69)	89 (41, 173)	164 (73, 401)
25-<35 years	2.2 (1.0, 4.2)	4.1 (1.4, 9.4)	7.6 (2.5, 19)	20 (9.1, 44)	80 (32, 187)	104 (38, 321)
35+ years	1.8 (0.9, 3.0)	4.7 (1.5, 10)	9.1 (2.9, 17)	22 (9.4, 46)	51 (23, 130)	117 (31, 375)
Maternal Race						
White	1.9 (0.9, 3.7)	3.0 (1.2, 8.3)	6.3 (2.0, 15)	16 (7.7, 37)	62 (25, 155)	76 (27, 208)
Non-White	2.9 (1.5, 5.3)	9.3 (4.5, 16)	19.2 (9.1, 38)	42 (21, 66)	102 (49, 203)	232 (104, 662)
Maternal Education						
High School or less	2.9 (1.5, 5.4)	9.2 (3.8, 17)	23 (9.9, 48)	42 (16, 74)	105 (49, 209)	231 (90, 558)
Some College	2.9 (1.4, 5.2)	6.6 (2.4, 13)	13 (6.5, 26)	32 (16, 50)	81 (40, 176)	164 (59, 458)
Completed College	1.7 (0.9, 3.3)	2.8 (1.2, 7.3)	12 (1.9, 11)	14 (7.1, 33)	57 (24, 149)	75 (25, 179)
Annual Income						
<$30,000	3.0 (1.5, 5.7)	8.7 (3.8, 15)	21 (9.9, 46)	42 (18, 73)	96 (44, 189)	232 (88, 727)
$30,000 - $75,000	2.0 (1.1, 4.0)	4.2 (1.5, 11)	8.4 (3.1, 19)	22 (10, 44)	79 (32, 189)	93 (38, 335)
≥$75,000	1.8 (0.8, 3.3)	3.1 (1.2, 7.2)	5.2 (1.7, 11)	15 (6.5, 35)	57 (24, 168)	84 (27, 207)
Maternal Smoking [a,b]						
Non-Smoking	2.1 (1.0, 4.3)	4.5 (1.5, 11)	8.7 (3.2, 22)	23 (9.5, 48)	75 (32, 169)	110 (38, 318)
Active Smoking	3.1 (1.7, 5.3)	8.6 (3.9, 16)	15 (6.6, 37)	36 (16, 73)	106 (41, 207)	267 (105, 596)
Parity [c]						
0	1.7 (0.9, 4.0)	3.7 (1.3, 8.9)	6.4 (1.9, 15)	18 (7.8, 43)	58 (22, 153)	93 (40, 278)
1	2.5 (1.3, 5.0)	6.7 (2.2, 13)	12 (4.7, 30)	26 (12, 57)	87 (40, 211)	114 (35, 324)
2 +	2.6 (1.1, 4.4)	7.0 (2.1, 15)	13 (5.3, 28)	32 (15.2, 57)	83 (36, 183)	193 (79, 567)
Pre-pregnancy BMI (kg/m2)						
Normal/Underweight <25	1.8 (0.8, 3.7)	3.5 (1.3, 9.0)	6.7 (2.2, 17)	18 (7.9, 43)	61 (27, 156)	91 (33, 302)
Overweight ≥25, <30	2.5 (1.1, 4.5)	5.4 (2.0, 12)	9.5 (4.2, 22)	24 (11, 49)	71 (28, 171)	130 (51, 348)
Obese ≥30	2.9 (1.6, 5.3)	8.6 (3.8, 15)	16 (7.5, 36)	42 (16, 74)	122 (55, 232)	161 (64, 504)
Child Sex						
Male	2.1 (1.1, 4.3)	5.2 (1.6, 11)	8.8 (3.6, 20)	23 (10, 49)	77 (34, 160)	116 (37, 340)
Female	2.2 (1.1, 4.6)	4.8 (1.7, 11)	9.9 (4.0, 25)	25 (10, 50)	76 (29, 184)	120 (49, 363)

BMI: body mass index, EARLI: Early Autism Risk Longitudinal Investigation Study, HOME: Health Outcomes and Measures of the Environment Study

[a] Maternal smoking during pregnancy for EARLI was based on maternal urinary cotinine concentrations (a metabolite of nicotine) during pregnancy. The cut off point of 50 ng/ml was used to differentiate between non-smoking and active smoking [62].

[b] Maternal smoking during pregnancy for HOME estimated based on maternal serum cotinine concentrations during pregnancy. The cut off point of 3.0 ng/ml was used to differentiate non-smoking and active smoking [61].

[c] Note that for parity, the EARLI cohort consists of mothers who had at least one previous child.

[d] Phthalate metabolites are ordered based on median concentration values from left (lowest median concentrations) the right (highest median concentrations).

Table S3. Univariate statistics of repeated maternal urinary concentrations of phthalate metabolites (ng/mL): the EARLI (2009–2012) and HOME Studies (2003–2006).

Phthalate Metabolite [a,b]		N	Percent>LOD	Minimum	Maximum	5th Percentile	25th Percentile	Median	75th Percentile	95th Percentile
MCPP										
	EARLI	280	6.43	0.28	2390.00	0.28	1.00	2.10	5.63	37.46
	HOME	552	4.76	0.14	66.7	0.30	1.10	3.36	4.40	9.50
MiBP										
	EARLI	280	1.07	0.57	527.00	1.50	4.70	9.35	16.83	35.95
	HOME	552	16.85	0.21	84.10	0.34	1.60	5.00	11.00	23.96
MBzP										
	EARLI	280	1.43	0.21	312.00	0.89	2.98	6.00	13.63	35.91
	HOME	552	3.00	0.16	820.80	0.75	3.74	9.36	23.40	80.35
MBP										
	EARLI	280	0.00	1.10	300.00	2.60	7.50	13.10	24.55	64.89
	HOME	552	0.00	0.80	2240	3.04	10.10	23.40	49.20	128.80
ΣDEHP [c]										
	EARLI	280	NA	1.77	2630.70	8.40	19.42	35.08	69.90	184.73
	HOME	552	NA	2.57	7211.43	9.57	31.93	77.07	175.387	840.44
MEP										
	EARLI	280	0.00	1.50	1330.00	4.39	13.78	26.95	62.35	213.30
	HOME	552	0.00	2.57	26004.00	11.56	43.69	117.48	351.78	1717.32

EARLI: Early Autism Risk Longitudinal Investigation Study, HOME: Health Outcomes and Measures of the Environment Study

[a] MCPP, mono(3-carboxypropyl) phthalate; MiBP, mono-isobutyl phthalate; MBzP, monobenzyl phthalate; MBP, mono-n-butyl phthalate; ΣDEHP, summary di(2-ethylhexyl) phthalate metabolite measure; MEP, monoethyl phthalate

[b] Phthalate metabolites are ordered based on median concentration values from left (lowest median concentrations) the right (highest median concentrations).

[c] Concentrations of ΣDEHP (in ng/mL) were calculated using the following formula: ΣDEHP (ng/mL) = [MECPP (ng/mL) /278 g/mol + MEHHP (ng/mL) /294.3 g/mol + MEOHP (ng/mL) /292.2 g/mol + MEHP (ng/mL) /278.3 g/mol] *278 g/mol.

Table S4. Pearson correlation coefficients between log-10 transformed gestational urinary phthalate metabolites (ng/mL)[a,b]: the EARLI (2009–2012) and HOME Studies (2003–2006).

	MBP	MBzP	MEP	MiBP	MCPP	ΣDEHP [c]
MBP	1.0	0.56	0.33	0.65	0.16	0.30
MBzP	0.44	1.0	0.16	0.40	0.00	0.18
MEP	0.15	0.12	1.0	0.21	-0.05	0.19
MiBP	0.48	0.36	0.27	1.0	0.15	0.24
MCPP	0.38	0.21	0.04	0.18	1.0	0.36
ΣDEHP	0.16	0.09	0.05	0.11	0.41	1.0
KEY	EARLI			HOME		

EARLI: Early Autism Risk Longitudinal Investigation Study, HOME: Health Outcomes and Measures of the Environment Study

[a] MCPP, mono(3-carboxypropyl) phthalate; MiBP, mono-isobutyl phthalate; MBzP, monobenzyl phthalate; MBP, mono-n-butyl phthalate; ΣDEHP, summary di(2-ethylhexyl) phthalate metabolite measure; MEP, monoethyl phthalate

[b] Phthalate metabolites are ordered based on median concentration values from left (lowest median concentrations) the right (highest median concentrations).

[c] Concentrations of ΣDEHP (in ng/mL) were calculated using the following formula: ΣDEHP (ng/mL) = [MECPP (ng/mL) /278 g/mol + MEHHP (ng/mL) /294.3 g/mol + MEOHP (ng/mL) /292.2 g/mol + MEHP (ng/mL) /278.3 g/mol] *278 g/mol.

Table S5. Intra-class correlation coefficients and (95% confidence intervals (CI)) for gestational urinary phthalate metabolites (ng/mL)[a,b] : the EARLI (2009–2012) and HOME Studies (2003–2006).

	EARLI	HOME
Time Period Collected	1st and 2nd or 3rd trimester	16 and 26 weeks gestation
Phthalate metabolite	ICC (95% CI)	ICC (95% CI)
MCPP	0.27 (0.11, 0.42)	0.29 (0.18, 0.39)
MiBP	0.54 (0.41, 0.65)	0.40 (0.30, 0.50)
MBzP	0.55 (0.42, 0.65)	0.49 (0.39, 0.57)
MBP	0.49 (0.35, 0.60)	0.25 (0.13, 0.36)
ΣDEHP [c]	0.03 (-0.13, 0.20)	0.16 (0.04, 0.27)
MEP	0.63 (0.52, 0.72)	0.42 (0.31, 0.51)

EARLI: Early Autism Risk Longitudinal Investigation Study, HOME: Health Outcomes and Measures of the Environment Study

[a] MCPP, mono(3-carboxypropyl) phthalate; MiBP, mono-isobutyl phthalate; MBzP, monobenzyl phthalate; MBP, mono-n-butyl phthalate; ΣDEHP, summary di(2-ethylhexyl) phthalate metabolite measure; MEP, monoethyl phthalate

[b] Phthalate metabolites are ordered based on median concentration values from left (lowest median concentrations) the right (highest median concentrations).

[c] Concentrations of ΣDEHP (in ng/mL) were calculated using the following formula: ΣDEHP (ng/mL) = [MECPP (ng/mL) /278 g/mol + MEHHP (ng/mL) /294.3 g/mol + MEOHP (ng/mL) /292.2 g/mol + MEHP (ng/mL) /278.3 g/mol] *278 g/mol.

Table S6: Unadjusted and adjusted [b] differences (95% Confidence Intervals (CI)) in Child SRS T-scores at 3 to 8 years per 10-fold increase in gestational urinary phthalate metabolite concentrations at quantiles of SRS T-scores[c]: the EARLI (2009–2012) and HOME Studies (2003–2006).

Phthalate Metabolite	Tau	EARLI (n=140) n [d]	EARLI Crude	EARLI Adjusted	HOME (n=276) n	HOME Crude	HOME Adjusted	POOLED Crude	POOLED Adjusted
MCPP	0.05	9	2 (-2, 2)	0 (-4, 2)	26	0 (-4, 0)	-1 (-6, 4)	0 (-3, 2)	1 (-3, 1)
	0.10	21	0 (0, 2)	0 (-1, 2)	35	2 (-3, 3)	0 (-4, 4)	0 (-1, 0)	1 (0, 1)
	0.25	41	0 (-4, 1)	-1 (-4, 1)	72	0 (-5, 4)	3 (-1, 7)	-2 (-4, -1)	1 (-2, 2)
	0.50	80	-3 (-4, -1)	-1 (-6, 0)	148	0 (-6, 4)	4 (-3, 7)	-4 (-6, -1)	0 (-2, 3)
	0.75	107	-4 (-6, 3)	-3 (-4, 2)	213	-1 (-7, 5)	4 (-1, 7)	-3 (-8, 3)	0 (-3, 2)
	0.90	127	-2 (-11, 5)	-2 (-10, 3)	249	-7 (-10, 5)	0 (-1, 15)	-4 (-10, 3)	0 (-4, 5)
	0.95	133	-9 (-14, 18)	-2 (-7, 17)	263	3 (-10, 17)	5 (-3, 15)	-3 (-8, 17)	-3 (-5, 2)
MiBP	0.05	9	-2 (-5, 6)	-2 (-9, 5)	26	0 (-2, 0)	1 (-1, 4)	-2 (-3, 0)	-1 (-2, 2)
	0.10	21	0 (-4, 1)	-1 (-7, 3)	35	0 (-2, 2)	1 (0, 3)	-2 (-3, 2)	0 (-1, 2)
	0.25	41	0 (-5, 6)	0 (-4, 6)	72	1 (-3, 3)	2 (0, 4)	-2 (-3, 1)	2 (-1, 3)
	0.50	80	-1 (-7, 3)	-1 (-6, 2)	148	4 (-1, 7)	2 (-1, 4)	-3 (-5, 1)	2 (-1, 3)
	0.75	107	-3 (-10, 3)	-7 (-11, 0)	213	4 (-1, 10)	-1 (-4, 4)	0 (-5, 4)	0 (-4, 3)
	0.90	127	-8 (-12, 7)	-4 (-10, 4)	249	12 (-1, 25)	1 (-4, 15)	3 (-8, 8)	0 (-4, 8)
	0.95	133	-2 (-15, 9)	-4 (-7, 5)	263	25 (4, 34)	14 (2, 23)	6 (-2, 23)	11 (1, 13)
MBzP	0.05	9	0 (-3, 2)	-1 (-2, 1)	26	0 (0, 0)	1 (-1, 3)	0 (0, 1)	0 (-2, 1)
	0.10	21	0 (-3, 0)	-1 (-2, 1)	35	1 (-3, 2)	1 (-1, 2)	0 (0, 1)	0 (-1, 1)
	0.25	41	-2 (-5, 3)	-2 (-5, 1)	72	4 (0, 6)	0 (-1, 3)	2 (-1, 4)	0 (-1, 2)
	0.50	80	-2 (-5, 4)	-1 (-5, 1)	148	5 (3, 9)	1 (-3, 4)	5 (4, 7)	1 (-2, 3)
	0.75	107	2 (-4, 7)	-1 (-4, 4)	213	6 (3, 8)	1 (-2, 5)	7 (3, 9)	1 (0, 6)
	0.90	127	9 (7, 19)	1 (-10, 10)	249	8 (2, 17)	5 (-2, 10)	10 (4, 16)	6 (1, 10)
	0.95	133	11 (4, 15)	3 (-9, 8)	263	20 (-2, 21)	10 (2, 14)	16 (1, 20)	10 (6, 11)
MBP	0.05	9	-4 (-5, 2)	-2 (-7, -1)	26	0 (-6, 0)	0 (-5, 4)	0 (-3, 0)	-2 (-5, 2)
	0.10	21	-2 (-8, 2)	-1 (-8, 1)	35	0 (-2, 2)	0 (-3, 3)	0 (-1, 0)	0 (-2, 1)
	0.25	41	-4 (-7, 1)	-3 (-7, 0)	72	1 (-1, 4)	0 (-1, 3)	0 (-2, 2)	0 (-2, 1)
	0.50	80	-4 (-9, -1)	-5 (-9, -1)	148	3 (-2, 7)	2 (-3, 3)	5 (2, 6)	-1 (-3, 2)
	0.75	107	3 (-13, 11)	-7 (-11, 5)	213	0 (-3, 6)	-2 (-5, 8)	3 (-2, 9)	-1 (-6, 5)
	0.90	127	0 (-14, 13)	-7 (-18, 4)	249	4 (-10, 13)	-1 (-4, 10)	5 (-7, 11)	-1 (-8, 3)
	0.95	133	-3 (-27, 25)	-12 (-15, 6)	263	2 (-11, 30)	14 (1, 16)	0 (-8, 25)	10 (-5, 14)
ΣDEHP [a]	0.05	9	0 (-7, 4)	1 (-5, 4)	26	0 (0, 2)	2 (-1, 3)	1 (-2, 3)	2 (0, 3)
	0.10	21	0 (-3, 3)	0 (-4, 4)	35	2 (-1, 2)	2 (0, 3)	2 (-1, 3)	2 (0, 3)
	0.25	41	0 (-8, 3)	-2 (-9, 1)	72	1 (-2, 3)	1 (0, 5)	3 (0, 4)	1 (0, 3)
	0.50	80	-4 (-7, -1)	-5 (-7, -1)	148	2 (-2, 6)	5 (2, 7)	4 (0, 6)	2 (0, 4)
	0.75	107	-7 (-12, -1)	-6 (-11, -2)	213	0 (-5, 5)	4 (-1, 7)	1 (-9, 5)	-1 (-4, 4)
	0.90	127	-19 (-27, -4)	-16 (-18, 7)	249	-5 (-9, 6)	5 (0, 11)	-7 (-10, 3)	2 (-3, 9)
	0.95	133	-15 (-34, -7)	-15 (-23, 9)	263	4 (-7, 14)	9 (-1, 17)	-5 (-8, 19)	8 (3, 13)
MEP	0.05	9	0 (-2, 0)	0 (-2, 0)	26	-2 (-3, 1)	-1 (-4, 1)	0 (-1, 0)	-1 (-2, 0)
	0.10	21	0 (-1, 0)	-1 (-2, 0)	35	-1 (-2, 1)	-1 (-3, 1)	0 (0, 1)	-1 (-1, 0)
	0.25	41	-2 (-2, 3)	-1 (-3, 0)	72	1 (-2, 3)	0 (-2, 2)	2 (-1, 3)	-1 (-2, 1)
	0.50	80	-2 (-6, 2)	-3 (-5, 1)	148	1 (-1, 3)	1 (-2, 3)	3 (1, 5)	0 (-2, 2)
	0.75	107	-3 (-8, 4)	-5 (-10, 2)	213	3 (-4, 7)	1 (-2, 3)	5 (1, 7)	0 (-4, 2)
	0.90	127	-12 (-14, 10)	-12 (-15, 6)	249	6 (-1, 12)	2 (-4, 9)	4 (-1, 8)	1 (-5, 7)
	0.95	133	-3 (-20, 23)	1 (-2, 16)	263	8 (2, 18)	3 (-6, 19)	6 (-2, 9)	1 (-6, 14)

EARLI: Early Autism Risk Longitudinal Investigation Study, HOME: Health Outcomes and Measures of the Environment Study, SRS: Social Responsiveness Scale

MCPP, mono(3-carboxypropyl) phthalate; MiBP, mono-isobutyl phthalate; MBzP, monobenzyl phthalate; MBP, mono-n-butyl phthalate; ΣDEHP, summary di(2-ethylhexyl) phthalate metabolite measure; MEP, monoethyl phthalate

Phthalate metabolites are ordered based on median concentration values from left (lowest median concentrations) the right (highest median concentrations).

[a] Concentrations of ΣDEHP (in ng/mL) were calculated using the following formula: ΣDEHP (ng/mL) = [MECPP (ng/mL) /278 g/mol + MEHHP (ng/mL) /294.3 g/mol + MEOHP (ng/mL) /292.2 g/mol + MEHP (ng/mL) /278.3 g/mol] *278 g/mol.

[b] Adjusted for maternal age (continuous), maternal race (white vs non-white), income (<$30,000 vs $30,000–$75,000, ≥ $75,000), parity (continuous), and $\log_{10}$–transformed urine/serum cotinine concentrations (continuous). Note cotinine concentrations were ascertained from maternal urine in EARLI and serum in HOME. In the pooled cohort model, adjusted for smoking during pregnancy as a binary variable. The pooled results are adjusted for cohort.

[c] Positive coefficients for SRS indicate that maternal phthalate exposure is associated with more deficits in social responsiveness traits

[d] Number of participants at each percentile of SRS scores

Table S7: Unadjusted and adjusted differences in children's SRS T-scores at ages 3 to 8 per 10-fold increase in gestational urinary phthalate metabolite concentrations using linear regression: the EARLI (2009–2012) and HOME Studies (2003–2006).

Phthalate Metabolite [c]		Linear regression Results: β (95% CI)					
		Full Sample		Male Children		Female Children	
		Crude	Adjusted	Crude	Adjusted	Crude	Adjusted
MCPP							
	EARLI	-3 (-7, 1)	-3 (-7, 1)	-2 (-8, 4)	-3 (-9, 3)	-3 (-7, 0)	-3 (-7, 0)
	HOME	0 (-5, 5)	3 (-1, 8)	4 (-3, 10)	4 (-2, 10)	-4 (-11, 3)	1 (-6, 8)
	POOLED	-3 (-6, 0)	0 (-3, 3)	0 (-5, 4)	0 (-4, 5)	-5 (-10, -1)	-1 (-5, 3)
MiBP							
	EARLI	-2 (-9, 4)	-2 (-8, 4)	-3 (-13, 7)	-2 (-11, 7)	-2 (-8, 5)	-1 (-7, 5)
	HOME	3 (0, 8)	3 (-1, 6)	5 (0, 10)	3 (-2, 8)	4 (-2, 10)	1 (-4, 7)
	POOLED	-1 (-4, 2)	1 (-2, 5)	1 (-3, 6)	1 (-3, 6)	-3 (-7, 1)	1 (-3, 5)
MBzP							
	EARLI	3 (-2, 8)	1 (-4, 6)	5 (-3, 14)	2 (-6, 10)	1 (-4, 6)	0 (-5, 5)
	HOME	5 (2, 8)	1 (-2, 5)	4 (0, 9)	1 (-3, 5)	5 (1, 10)	1 (-4, 6)
	POOLED	5 (3, 8)	2 (-1, 4)	5 (1, 9)	2 (-2, 6)	6 (2, 9)	1 (-2, 5)
MBP							
	EARLI	-2 (-9, 4)	-4 (-10, 3)	-3 (-13, 8)	-5 (-14, 5)	-1 (-8, 6)	-2 (-9, 4)
	HOME	2 (-3, 7)	1 (-4, 5)	4 (-3, 10)	1 (-5, 7)	0 (-6, 7)	-2 (-8, 4)
	POOLED	3 (-1, 6)	0 (-4, 3)	1 (-4, 7)	-1 (-6, 4)	4 (-1, 9)	-1 (-6, 4)
ΣDEHP							
	EARLI	-7 (-14, 0)	-7 (-14, -1)	-7 (-18, 3)	-7 (-17, 2)	-5 (-11, 2)	-6 (-13, 0)
	HOME	1 (-2, 4)	4 (1, 7)	1 (-4, 5)	3 (-1, 7)	1 (-4, 6)	4 (-1, 8)
	POOLED	1 (-1, 4)	1 (-2, 4)	-1 (-5, 3)	1 (-3, 5)	3 (-1, 7)	2 (-2, 6)
MEP							
	EARLI	-2 (-6, 2)	-3 (-7, 1)	-2 (-9, 5)	-3 (-9, 3)	-2 (-6, 3)	-3 (-8, 1)
	HOME	2 (0, 5)	1 (-2, 3)	2 (-2, 5)	1 (-3, 4)	4 (1, 6)	1 (-3, 5)
	POOLED	3 (1, 4)	0 (-2, 2)	1 (-2, 3)	-1 (-4, 2)	5 (2, 7)	0 (-3, 3)

EARLI: Early Autism Risk Longitudinal Investigation Study, HOME: Health Outcomes and Measures of the Environment Study, SRS: Social Responsiveness Scale

[a] Adjusted for maternal age (continuous), maternal race (white vs non-white), income (<\$30,000 vs \$30,000–\$75,000, ≥ \$75,000), parity (continuous), and log_{10}–transformed urine/serum cotinine concentrations (continuous). Note cotinine concentrations were ascertained from maternal urine in EARLI and serum in HOME. In the pooled cohort model, adjusted for smoking during pregnancy as a binary variable. The pooled results are adjusted for cohort.

[b] Positive coefficients for SRS indicate that maternal phthalate exposure is associated with more deficits in social responsiveness traits.

[c] Phthalate metabolites are ordered based on median concentration values from left (lowest median concentrations) the right (highest median concentrations).

Table S8. Unadjusted and adjusted differences in children's SRS T-scores at ages 3 to 8 per 10-fold increase in gestational urinary phthalate metabolite concentrations using quantile regression, stratified by child sex: the EARLI (2009–2012) and HOME Studies (2003–2006).

Phthalate Metabolite	Tau	Quantile Regression Results: β (95% CI)			
		EARLI		HOME	
		Male Children	Female Children	Male Children	Female Children
MCPP	0.05	2 (-13, 9)	-1 (-11, 14)	-3 (-10, 11)	6 (-7, 15)
	0.10	2 (1, 7)	-1 (-5, 1)	-3 (-11, 4)	2 (-9, 12)
	0.25	2 (-4, 6)	-1 (-6, 0)	1 (-5, 8)	2 (-4, 8)
	0.50	-1 (-12, 2)	-2 (-5, -1)	7 (-5, 12)	2 (-5, 7)
	0.75	-2 (-6, 3)	-3 (-7, -3)	3 (-3, 14)	0 (-11, 8)
	0.90	0 (-8, 11)	-2 (-6, 11)	9 (0, 19)	-4 (-12, 6)
	0.95	-2 (-11, 27)	-8 (-14, 19)	17 (-2, 24)	-3 (-12, 0)
MiBP	0.05	2 (-17, 8)	0 (-8, 12)	2 (-2, 10)	5 (-4, 5)
	0.10	1 (-7, 15)	2 (-8, 11)	3 (-2, 7)	1 (-4, 7)
	0.25	2 (-4, 12)	2 (-5, 8)	2 (-2, 6)	1 (-1, 3)
	0.50	-3 (-10, 9)	1 (-7, 6)	2 (-5, 6)	1 (-3, 4)
	0.75	-10 (-14, 1)	1 (-9, 2)	-2 (-5, 6)	0 (-9, 6)
	0.90	-5 (-13, 1)	-2 (-6, 11)	1 (-1, 14)	4 (-11, 21)
	0.95	-5 (-7, 4)	-11 (-11, 32)	10 (-15, 26)	8 (-18, 25)
MBzP	0.05	0 (-13, 2)	0 (-6, 6)	2 (1, 2)	-4 (-7, 6)
	0.10	-2 (-3, 4)	1 (-4, 3)	2 (0, 4)	-1 (-7, 4)
	0.25	0 (-5, 5)	0 (-5, 2)	0 (-1, 4)	-1 (-6, 3)
	0.50	0 (-8, 6)	-1 (-5, 3)	-2 (-5, 4)	1 (-5, 5)
	0.75	3 (-9, 9)	0 (-5, 4)	-3 (-7, 6)	3 (-1, 7)
	0.90	1 (-9, 6)	2 (-6, 8)	2 (-9, 10)	5 (-2, 8)
	0.95	5 (-1, 8)	4 (-15, 17)	14 (-20, 17)	4 (-2, 14)
MBP	0.05	-2 (-9, 3)	-1 (-11, 2)	0 (-6, 6)	-1 (-8, 4)
	0.10	-2 (-8, 2)	-1 (-8, 1)	0 (-4, 3)	1 (-6, 4)
	0.25	-3 (-8, 2)	-3 (-5, 1)	0 (-3, 3)	1 (-2, 3)
	0.50	-8 (-17, 4)	-4 (-7, 2)	2 (-5, 7)	-1 (-5, 3)
	0.75	-13 (-23,1)	-6 (-10, 3)	-1 (-6, 9)	-4 (-10, 4)
	0.90	-11 (-28, -1)	-3 (-5, 14)	2 (-1, 6)	1 (-12, 16)
	0.95	-12 (-28, 8)	8 (-10, 32)	11 (-17, 14)	4 (-11, 15)
ΣDEHP	0.05	3 (-13, 4)	-1 (-24, 2)	2 (-3, 4)	4 (-5, 7)
	0.10	3 (-8, 4)	-1 (-14, 2)	2 (-1, 4)	4 (-1, 7)
	0.25	1 (-8, 6)	-3 (-11, 0)	1 (-1, 5)	4 (0, 5)
	0.50	-7 (-10, 3)	-5 (-9, -1)	4 (-1, 9)	3 (0, 6)
	0.75	-7 (-16, 0)	-6 (-13, -2)	2 (-3, 10)	4 (-2, 13)
	0.90	-5 (-21, 8)	-6 (-14, 6)	2 (-1, 12)	7 (-6, 11)
	0.95	-9 (-15, 10)	-11 (-27, 17)	8 (-7, 21)	0 (-4, 22)
MEP	0.05	0 (-1, 1)	0 (-1, 1)	0 (-7, 5)	-1 (-6, 0)
	0.10	0 (-4, 1)	1 (-1, 2)	1 (-4, 4)	-2 (-4, 2)
	0.25	-2 (-5, 0)	1 (0, 2)	0 (-2, 3)	-2 (-3, 1)
	0.50	-6 (-8, 2)	0 (-4, 3)	1 (-3, 4)	1 (-1, 4)
	0.75	-9 (-15, 8)	-6 (-8, 4)	2 (-3, 4)	0 (-5, 6)
	0.90	0 (-16, 15)	-11 (-14, 5)	1 (-1, 7)	3 (-6, 12)
	0.95	4 (-19, 18)	-11 (-20, 11)	10 (-4, 19)	-1 (-4, 18)

EARLI: Early Autism Risk Longitudinal Investigation Study, HOME: Health Outcomes and Measures of the Environment Study, SRS: Social Responsiveness Scale

MCPP, mono(3-carboxypropyl) phthalate; MiBP, mono-isobutyl phthalate; MBzP, monobenzyl phthalate; MBP, mono-n-butyl phthalate; ΣDEHP, summary di(2-ethylhexyl) phthalate metabolite measure; MEP, monoethyl phthalate

Phthalate metabolites are ordered based on median concentration values from left (lowest median concentrations) the right (highest median concentrations).

[a] Concentrations of ΣDEHP (in ng/mL) were calculated using the following formula: ΣDEHP (ng/mL) = [MECPP (ng/mL) /278 g/mol + MEHHP (ng/mL) /294.3 g/mol + MEOHP (ng/mL) /292.2 g/mol + MEHP (ng/mL) /278.3 g/mol] *278 g/mol.

[b] Adjusted for maternal age (continuous), maternal race (white vs non-white), income (<$30,000 vs $30,000–$75,000, ≥ $75,000), parity (continuous), and $\log_{10}$–transformed urine/serum cotinine concentrations (continuous). Note cotinine concentrations were ascertained from maternal urine in EARLI and serum in HOME. In the pooled cohort model, adjusted for smoking during pregnancy as a binary variable. The pooled results are adjusted for cohort.

[c] Positive coefficients for SRS indicate that maternal phthalate exposure is associated with more deficits in social responsiveness traits.

Table S9: Unadjusted and adjusted [b, c] differences (95% confidence intervals (CI)) in child SRS raw scores at 3 to 8 years per 10-fold increase in gestational urinary phthalate metabolite concentrations at quantiles of SRS T-scores: [d] the EARLI (2009–2012) and HOME Studies (2003–2006).

Phthalate		EARLI		HOME	
Metabolite	Tau	SRS T-scores	SRS raw scores	SRS T-scores	SRS raw scores
MCPP	0.05	0 (-4, 2)	-1 (-9, 7)	-1 (-6, 4)	-1 (-6, 8)
	0.10	0 (-1, 2)	1 (-3, 4)	0 (-4, 4)	3 (-4, 8)
	0.25	-1 (-4, 1)	-3 (-10, 4)	3 (-1, 7)	1 (-2, 12)
	0.50	-1 (-6, 0)	-10 (-15, 0)	4 (-3, 7)	7 (-6, 14)
	0.75	-3 (-4, 2)	-10 (-15, 2)	4 (-1, 7)	10 (1, 11)
	0.90	-2 (-10, 3)	-9 (-10, 29)	0 (-1, 15)	8 (-3, 20)
	0.95	-2 (-7, 17)	-11 (-15, 43)	5 (-3, 15)	11 (-2, 31)
	Mean [e]	-3 (-7, 1)	-7 (-18, 3)	3 (-1, 8)	7 (-2, 16)
MiBP	0.05	-2 (-9, 5)	-9 (-26, 17)	1 (-1, 4)	3 (-2, 6)
	0.10	-1 (-7, 3)	-4 (-19, 8)	1 (0, 3)	4 (-1, 7)
	0.25	0 (-4, 6)	3 (-15, 16)	2 (0, 4)	2 (1, 8)
	0.50	-1 (-6, 2)	-4 (-19, 7)	2 (-1, 4)	4 (-3, 9)
	0.75	-7 (-11, 0)	-18 (-30, 3)	-1 (-4, 4)	-1 (-9, 7)
	0.90	-4 (-10, 4)	-1 (-22, 9)	1 (-4, 15)	6 (-3, 19)
	0.95	-4 (-7, 5)	-7 (-11, 15)	14 (2, 23)	20 (4, 46)
	mean	-2 (-8, 4)	-6 (-21, 9)	3 (-1, 6)	6 (-1, 14)
MBzP	0.05	-1 (-2, 1)	-3 (-8, 2)	1 (-1, 3)	1 (-1, 5)
	0.10	-1 (-2, 1)	-2 (-3, 0)	1 (-1, 2)	2 (-3, 4)
	0.25	-2 (-5, 1)	-5 (-11, 4)	0 (-1, 3)	0 (-2, 7)
	0.50	-1 (-5, 1)	-3 (-14, 5)	1 (-3, 4)	3 (-3, 7)
	0.75	-1 (-4, 4)	-6 (-15, 8)	1 (-2, 5)	0 (-5, 6)
	0.90	1 (-10, 10)	-3 (-12, 10)	5 (-2, 10)	6 (-4, 16)
	0.95	3 (-9, 8)	1 (-10, 17)	10 (2, 14)	13 (-2, 19)
	mean	1 (-4, 6)	2 (-11, 14)	1 (-4, 5)	3 (-3, 9)
MBP	0.05	-2 (-7, -1)	-7 (-13, -1)	0 (-5, 4)	2 (-3, 5)
	0.10	-1 (-8, 1)	-3 (-15, 0)	0 (-3, 3)	3 (-6, 6)
	0.25	-3 (-7, 0)	-7 (-18, -3)	0 (-1, 3)	0 (-3, 3)
	0.50	-5 (-9, -1)	-7 (-22, 4)	2 (-3, 3)	3 (-5, 7)
	0.75	-7 (-11, 5)	-20 (-35, -7)	-2 (-5, 8)	-6 (-12, 5)
	0.90	-7 (-18, 4)	-14 (-27, 8)	-1 (-4, 10)	-1 (-12, 14)
	0.95	-12 (-15, 6)	-11 (-28, 23)	14 (1, 16)	3 (-10, 26)
	mean	-4 (-10, 3)	-9 (-24, 7)	1 (-4, 5)	1 (-7, 9)
ΣDEHP [a]	0.05	1 (-5, 4)	3 (-17, 9)	2 (-1, 3)	4 (-3, 7)
	0.10	0 (-4, 4)	-1 (-10, 10)	2 (0, 3)	3 (1, 6)
	0.25	-2 (-9, 1)	-2 (-23, 9)	1 (0, 5)	2 (0, 8)
	0.50	-5 (-7, -1)	-13 (-20, -1)	5 (2, 7)	7 (3, 13)
	0.75	-6 (-11, -2)	-23 (-33, -3)	4 (-1, 7)	8 (-3, 13)
	0.90	-16 (-18, 7)	-11 (-45, 13)	5 (0, 11)	9 (5, 18)
	0.95	-15 (-23, 9)	-16 (-67, 26)	9 (-1, 17)	14 (-1, 24)
	mean	-7 (-14, -1)	-17 (-33, -15)	1 (-4, 5)	7 (1, 13)
MEP	0.05	0 (-2, 0)	-1 (-3, 0)	-1 (-4, 1)	-3 (-5, 3)
	0.10	-1 (-2, 0)	-2 (-4, 0)	-1 (-3, 1)	-2 (-5, 4)
	0.25	-1 (-3, 0)	-3 (-8, 1)	0 (-2, 2)	0 (-3, 2)
	0.50	-3 (-5, 1)	-2 (-16, 5)	1 (-2, 3)	2 (-5, 7)
	0.75	-5 (-10, 2)	-3 (-22, 11)	1 (-2, 3)	0 (-3, 4)
	0.90	-12 (-15, 6)	-17 (-41, 12)	2 (-4, 9)	5 (-5, 13)
	0.95	1 (-2, 16)	-20 (-40, 19)	3 (-6, 19)	3 (-8, 27)
	mean	-3 (-7, 1)	-7 (-17, 4)	1 (-2, 3)	1 (-4, 6)

EARLI: Early Autism Risk Longitudinal Investigation Study, HOME: Health Outcomes and Measures of the Environment Study, SRS: Social Responsiveness Scale
MCPP, mono(3-carboxypropyl) phthalate; MiBP, mono-isobutyl phthalate; MBzP, monobenzyl phthalate; MBP, mono-n-butyl phthalate; ΣDEHP, summary di(2-ethylhexyl) phthalate metabolite measure; MEP, monoethyl phthalate
Phthalate metabolites are ordered based on median concentration values from left (lowest median concentrations) the right (highest median concentrations).
[a] Concentrations of ΣDEHP (in ng/mL) were calculated using the following formula: ΣDEHP (ng/mL) = [MECPP (ng/mL) /278 g/mol + MEHHP (ng/mL) /294.3 g/mol + MEOHP (ng/mL) /292.2 g/mol + MEHP (ng/mL) /278.3 g/mol] *278 g/mol.
[b] Adjusted for maternal age (continuous), maternal race (white vs non-white), income (<$30,000 vs $30,000–$75,000, ≥ $75,000), parity (continuous), and $\log_{10}$–transformed urine/serum cotinine concentrations (continuous). Note cotinine concentrations were ascertained from maternal urine in EARLI and serum in HOME.
[c] Adjusted for maternal age (continuous), maternal race (white vs non-white), income (<$30,000 vs $30,000–$75,000, ≥ $75,000), parity (continuous), $\log_{10}$–transformed urine/serum cotinine concentrations (continuous), child sex, and child age. Note cotinine concentrations were ascertained from maternal urine in EARLI and serum in HOME.
[d] Positive coefficients for SRS indicate that maternal phthalate exposure is associated with more deficits in social responsiveness traits
[e] Results from linear regression analyses

Table S10: Adjusted differences (95% confidence intervals (CI)) in child SRS T-scores at 3 to 8 years per 10-fold increase in gestational urinary monobenzyl phthalate (MBzP) metabolite concentrations at quantiles of SRS T-scores [c]: the EARLI (2009–2012) and HOME Studies (2003–2006).

	Quantile Regression Results: β (95% CI)						
	Tau	Primary Analysis	Adjusting for Child Sex	Adjusting for Child Age (Years)	Adjusting for Pre-pregnancy BMI	Adjusting for MBP	Adjusting for NICU admittance [d]
EARLI							
	0.05	-1 (-2, 1)	-1 (-2, 1)	-1 (-2, 1)	-1 (-3, 1)	0 (-2, 2)	
	0.10	-1 (-2, 1)	-1 (-2, 1)	0 (-2, 6)	-1 (-4, 1)	0 (-1, 2)	
	0.25	-2 (-5, 1)	-2 (-5, 2)	-2 (-5, 2)	-2 (-4, 2)	1 (-4, 3)	
	0.50	-1 (-5, 1)	-2 (-4, 2)	-2 (-5, 0)	-2 (-4, 2)	1 (-3, 3)	
	0.75	-1 (-4, 4)	-1 (-4, 4)	-2 (-5, 3)	0 (-7, 3)	2 (-3, 8)	
	0.90	1 (-10, 10)	0 (-5, 4)	2 (-10, 10)	0 (-10, 10)	14 (-8, 20)	
	0.95	3 (-9, 8)	2 (-3, 7)	3 (-6, 5)	0 (-7, 7)	13 (-7, 22)	
HOME							
	0.05	1 (-1, 3)	1 (-1, 2)	0 (-2, 2)	1 (-1, 2)	1 (-2, 3)	1 (-2, 2)
	0.10	1 (-1, 2)	1 (-1, 2)	1 (-1, 1)	1 (-1, 2)	1 (0, 2)	1 (-3, 2)
	0.25	0 (-1, 3)	0 (-1, 3)	0 (-1, 4)	1 (-2, 3)	0 (-2, 4)	1 (-2, 3)
	0.50	1 (-3, 4)	1 (-2, 4)	1 (-2, 4)	1 (-2, 4)	2 (-3, 5)	1 (-3, 3)
	0.75	1 (-2, 5)	0 (-3, 4)	1 (-3, 3)	1 (-2, 3)	1 (-2, 6)	0 (-3, 3)
	0.90	5 (-2, 10)	4 (0, 8)	4 (0, 10)	5 (-2, 9)	5 (0, 9)	5 (-2, 11)
	0.95	10 (2, 14)	9 (4, 13)	7 (-2, 11)	7 (6, 15)	7 (2, 12)	10 (3, 14)

EARLI: Early Autism Risk Longitudinal Investigation Study, HOME: Health Outcomes and Measures of the Environment Study, SRS: Social Responsiveness Scale, BMI: body mass index, MBzP: monobenzyl phthalate, MBP: mono-n-butyl phthalate, NICU: Neonatal Intensive Care Unit

[a] Concentrations of ΣDEHP (in ng/mL) were calculated using the following formula: ΣDEHP (ng/mL) = [MECPP (ng/mL) /278 g/mol + MEHHP (ng/mL) /294.3 g/mol + MEOHP (ng/mL) /292.2 g/mol + MEHP (ng/mL) /278.3 g/mol] *278 g/mol.

[b] Adjusted for maternal age (continuous), maternal race (white vs non-white), income (<$30,000 vs $30,000–$75,000, ≥ $75,000), parity (continuous), and $\log_{10}$–transformed urine/serum cotinine concentrations (continuous). Note cotinine concentrations were ascertained from maternal urine in EARLI and serum in HOME. In the pooled cohort model, adjusted for smoking during pregnancy as a binary variable. The pooled results are adjusted for cohort.

[c] Positive coefficients for SRS indicate that maternal phthalate exposure is associated with more deficits in social responsiveness traits.

[d] NICU admittance information was only available for a subset of participants (n= 265) in the HOME Study only, of which only n = 14 (5%) of HOME Study participants were admitted to the NICU

Figure S1. Flow chart of participant selection to final sample size

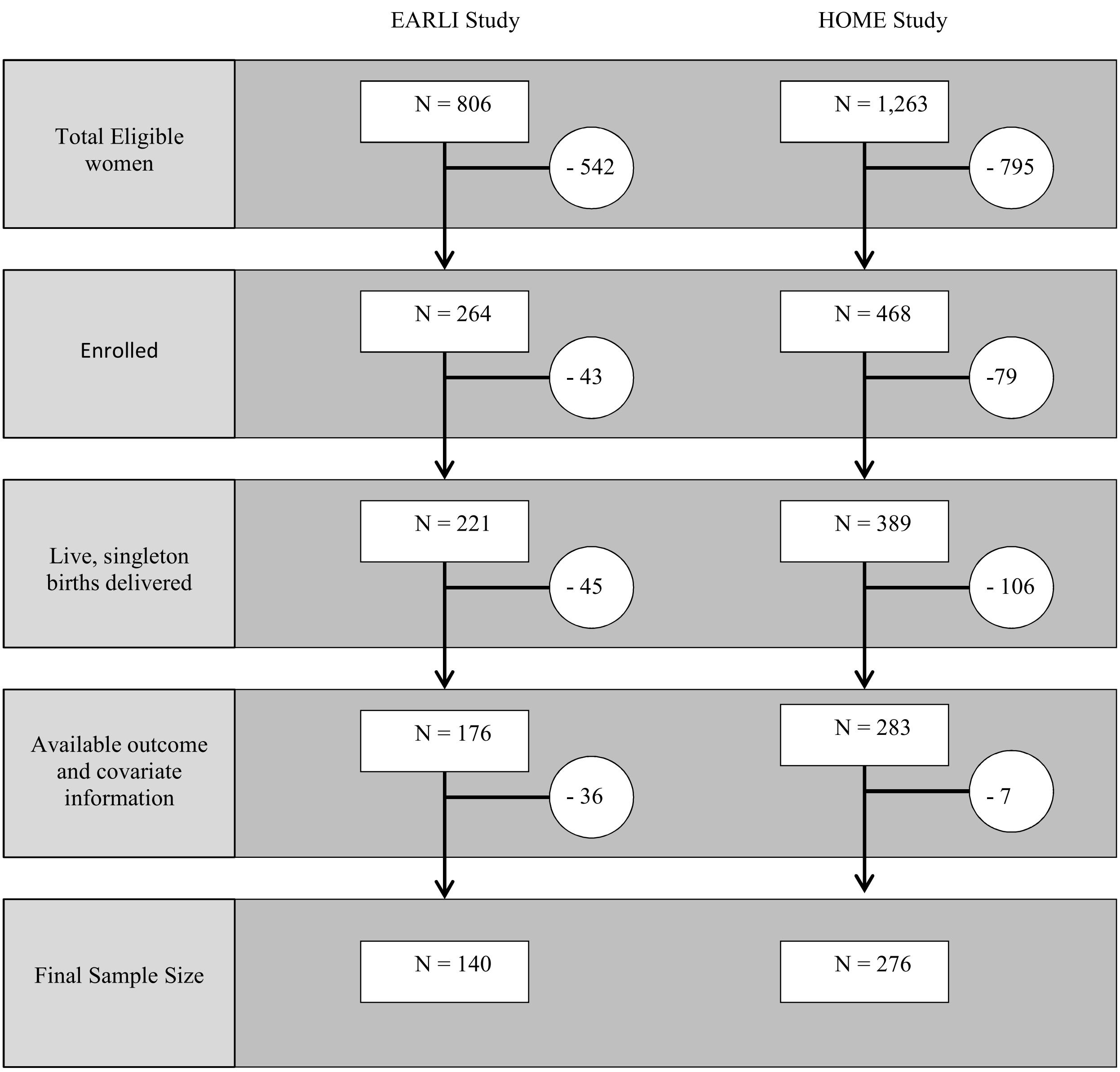

Figure S2. Directed acyclic graph used to select covariates for our primary analysis in the association between maternal urinary phthalate concentrations during gestation and child SRS scores

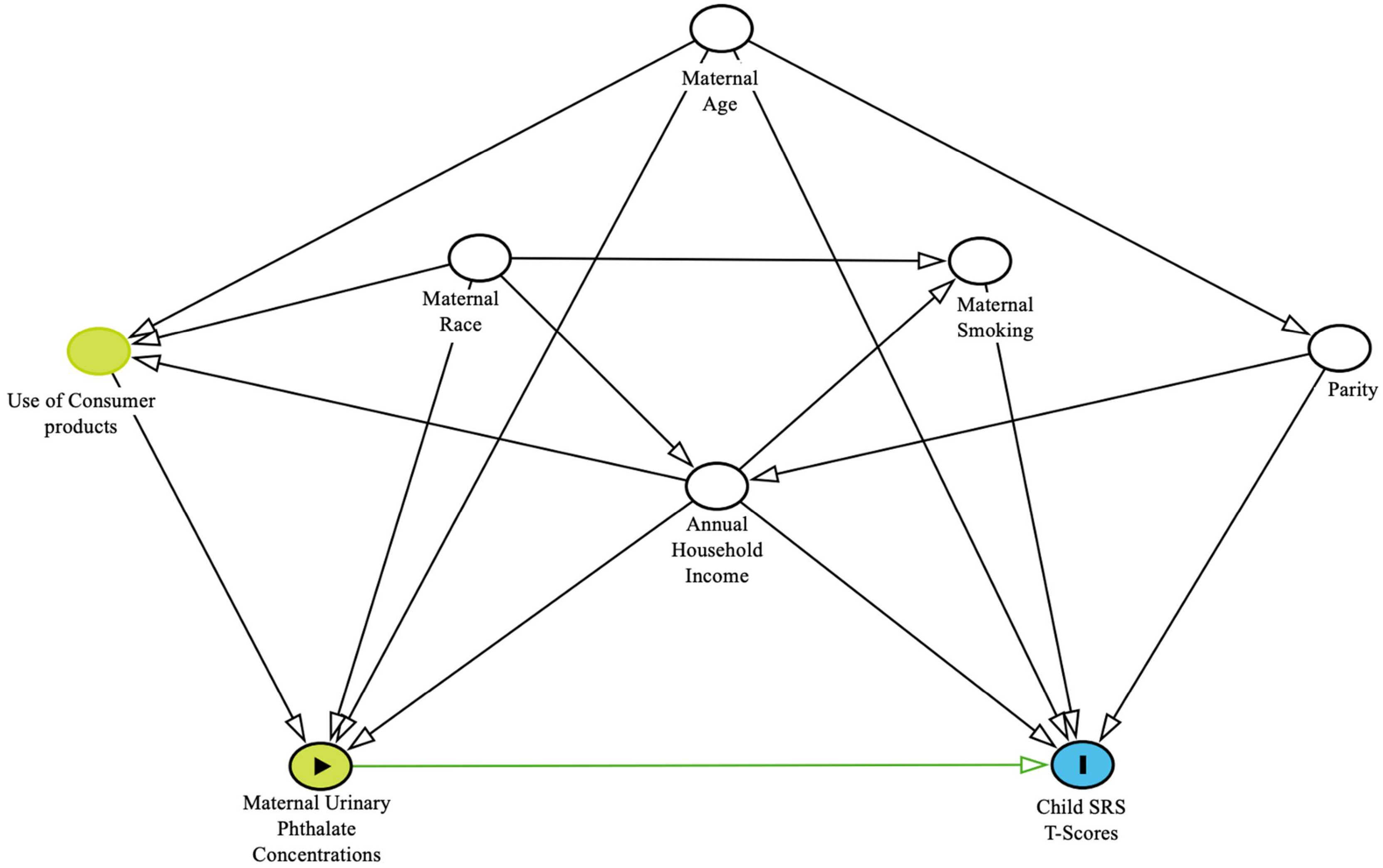

Figure S3. Directed acyclic graph used to select covariates for our primary and secondary analysis in the association between maternal urinary phthalate concentrations during gestation and child SRS scores

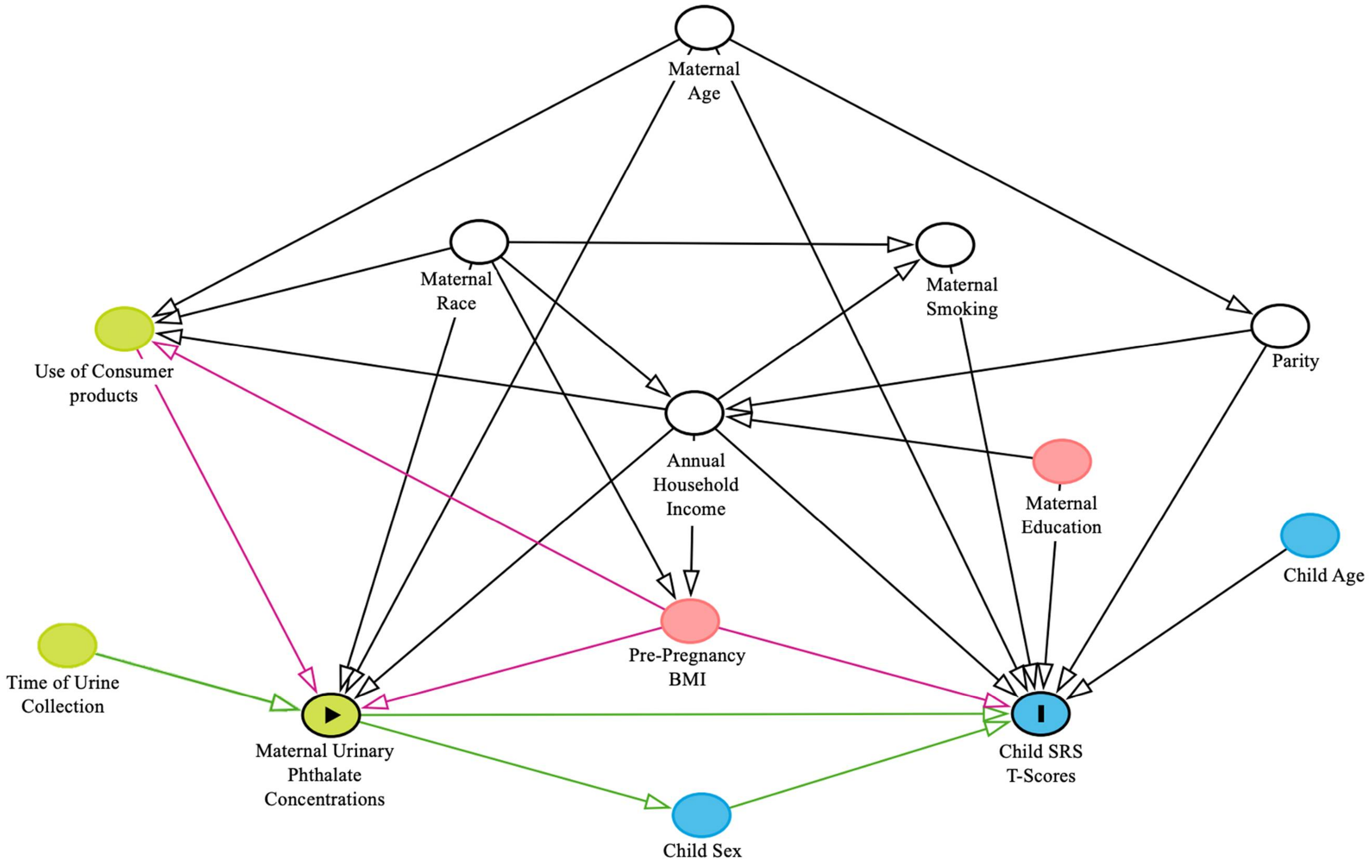

Figure S4. Kernel density plot of distributions of Children's SRS T-Score at Ages 3 to 8: the EARLI (2009–2012) and HOME Studies (2003–2006).

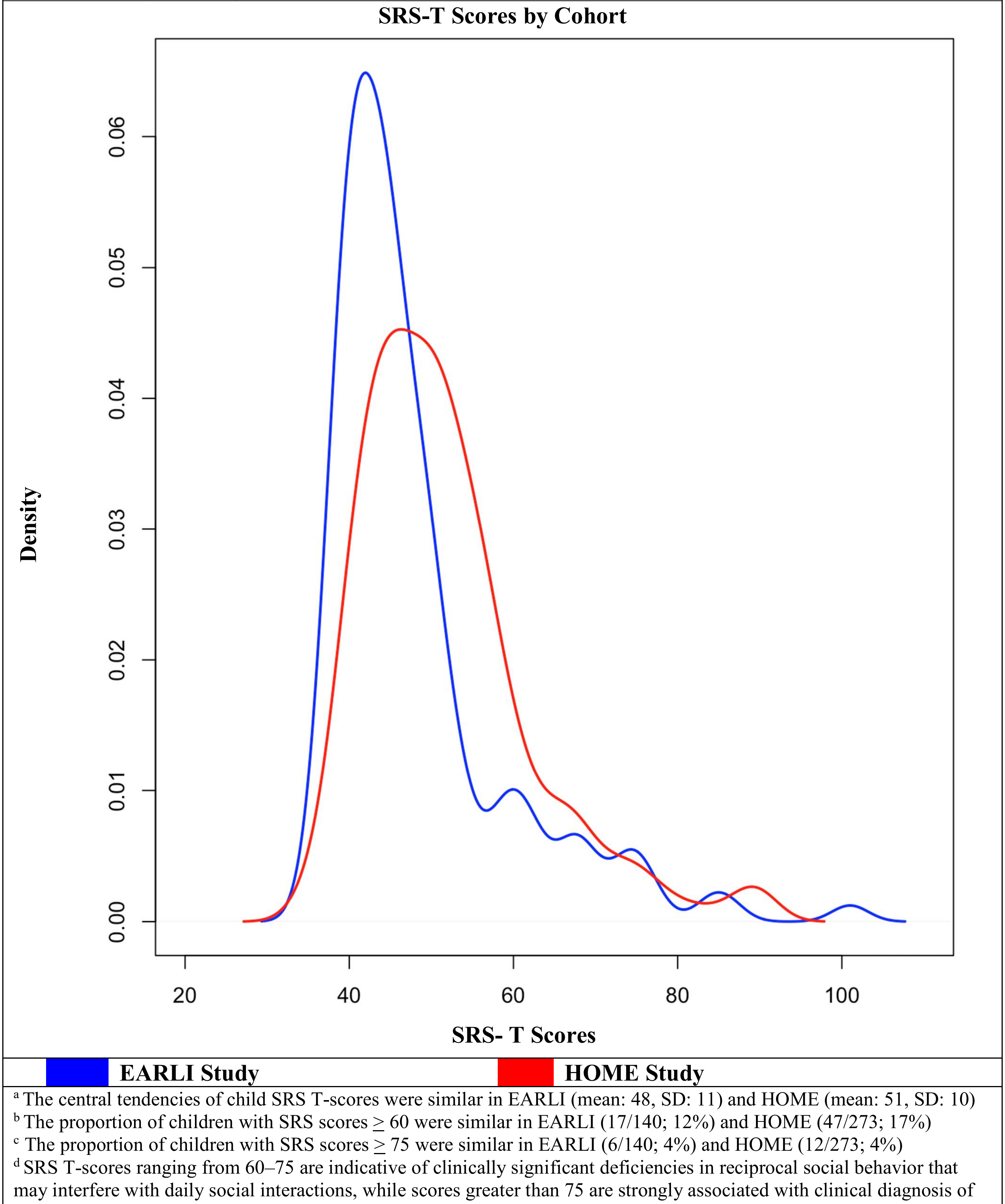

[a] The central tendencies of child SRS T-scores were similar in EARLI (mean: 48, SD: 11) and HOME (mean: 51, SD: 10)
[b] The proportion of children with SRS scores $\geq$ 60 were similar in EARLI (17/140; 12%) and HOME (47/273; 17%)
[c] The proportion of children with SRS scores $\geq$ 75 were similar in EARLI (6/140; 4%) and HOME (12/273; 4%)
[d] SRS T-scores ranging from 60–75 are indicative of clinically significant deficiencies in reciprocal social behavior that may interfere with daily social interactions, while scores greater than 75 are strongly associated with clinical diagnosis of ASD

Figure S5. Adjusted differences in children's SRS T-scores at ages 3 to 8 per 10-fold increase in gestational urinary phthalate metabolite concentrations using quantile regression: the EARLI (2009–2012) and HOME Studies (2003–2006).

EARLI	HOME

MCPP

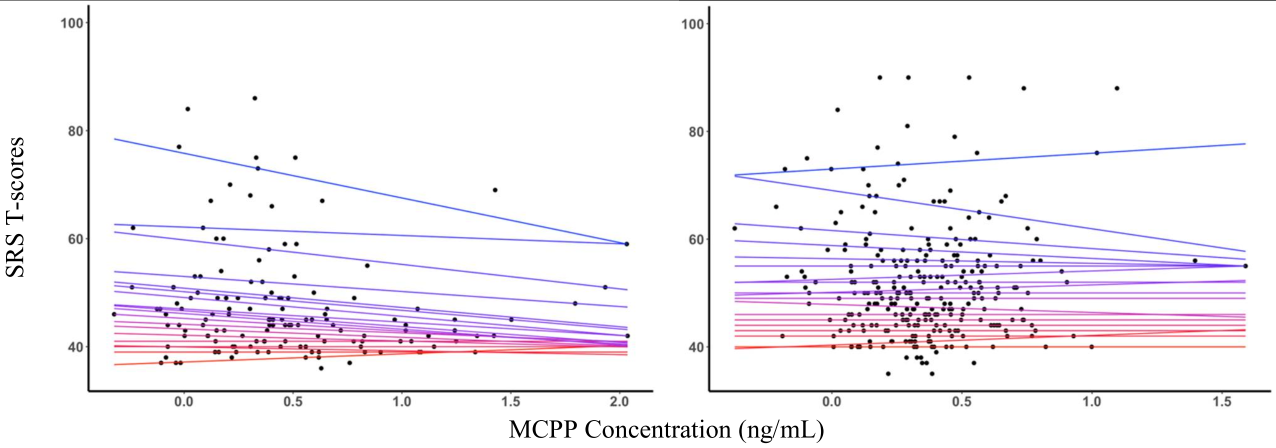

MiBP

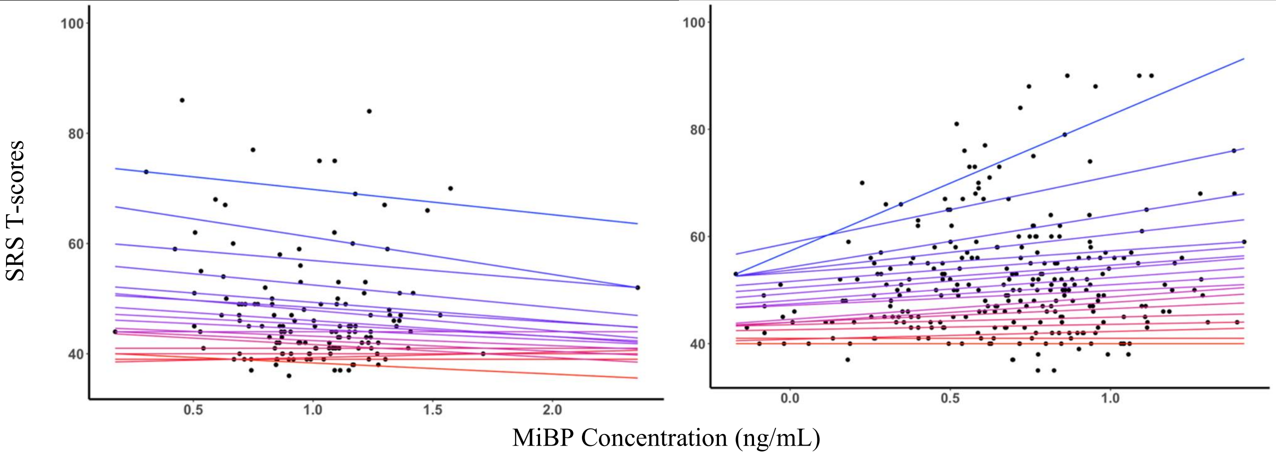

MBzP

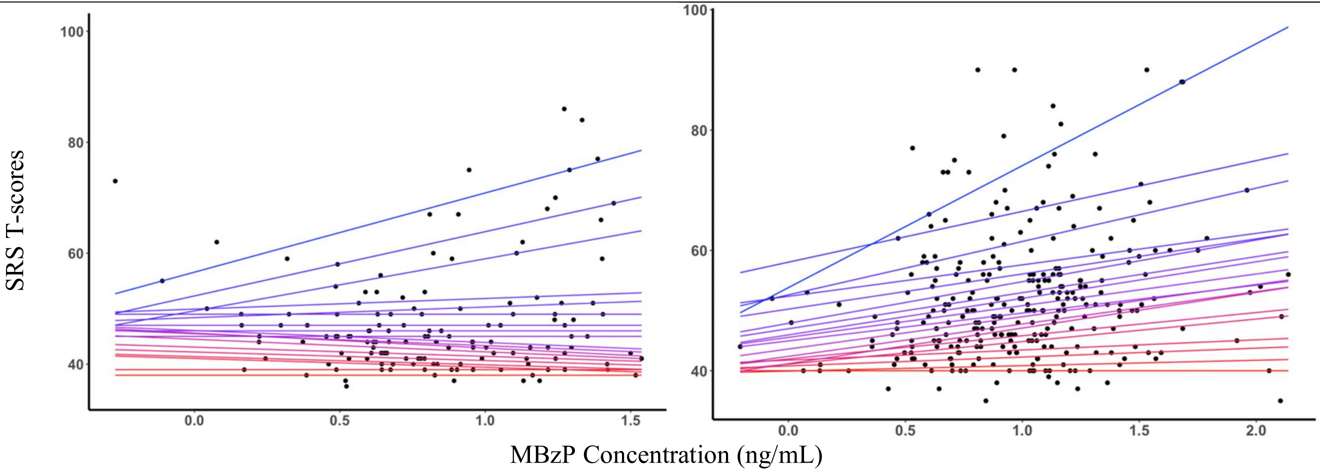

Figure S5. Continued

EARLI	HOME

MBP

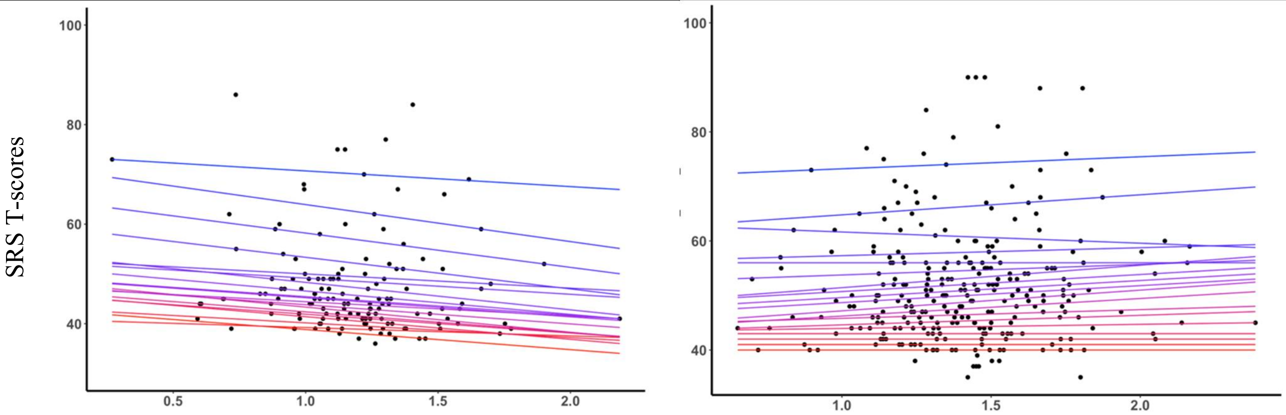

MBP Concentration (ng/mL))

ΣDEHP

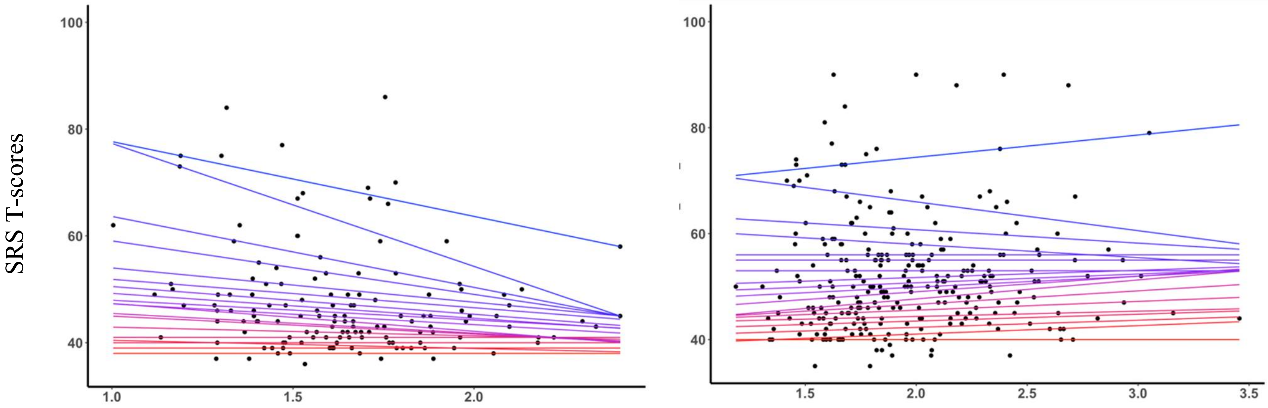

ΣDEHP Concentration (ng/mL)

MEP

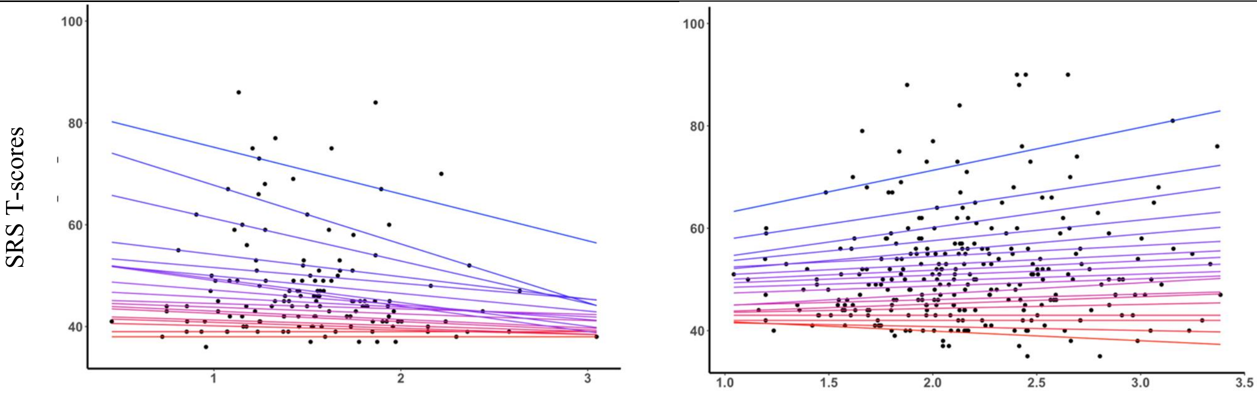

MEP Concentration (ng/mL)

Quantile	*0.25* *0.50* *0.75*

EARLI: Early Autism Risk Longitudinal Investigation Study, HOME: Health Outcomes and Measures of the Environment Study, SRS: Social Responsiveness Scale, MCPP: mono(3-carboxypropyl) phthalate; MiBP: mono-isobutyl phthalate; MBzP: monobenzyl phthalate; MBP: mono-n-butyl phthalate; ΣDEHP: summary di(2-ethylhexyl) phthalate metabolite measure; MEP, monoethyl phthalate

Phthalate metabolites are ordered based on median concentration values from left (lowest median concentrations) the right (highest median concentrations).

[a] Concentrations of ΣDEHP (in ng/mL) were calculated using the following formula: ΣDEHP (ng/mL) = [MECPP (ng/mL) /278 g/mol + MEHHP (ng/mL) /294.3 g/mol + MEOHP (ng/mL) /292.2 g/mol + MEHP (ng/mL) /278.3 g/mol] *278 g/mol.

[b] Adjusted for maternal age (continuous), maternal race (white vs non-white), income (<$30,000 vs $30,000–$75,000, ≥ $75,000), parity (continuous), and log_{10} –transformed urine/serum cotinine concentrations (continuous). Note cotinine concentrations were ascertained from maternal urine in EARLI and serum in HOME.

[c] Positive coefficients for SRS indicate that maternal phthalate exposure is associated with more deficits in social responsiveness traits

[d] Quantile sequence 0.05–0.95 by 0.05
